# What and when: a Delphi study identifying AI competencies and learning objectives for medical students

**DOI:** 10.3389/fdgth.2026.1918847

**Published:** 2026-09-30

**Authors:** Jennifer Simoni, José Luis Pereira, Sofía Crespo Sánchez, Diego Egaña-Yin, Carlota García-Gargallo, Oliver L. A. Hertog, Andrés Idoate, Lourdes López-Ortiz, Ian Morales-Gutiérrez, Alexandra Romero, Paula Santiago-Martínez, Gabriela Sawczyn, Adrián Vahamaki, Judith Urtubia-Fernandez, Elisa Mengual

**Affiliations:** 1 Medical Education Unit, University of Navarra School of Medicine, Pamplona, Spain; 2 Institute of Culture and Society, University of Navarra, Pamplona, Spain; 3 University of Navarra School of Medicine, Pamplona, Spain; 4 Department of Pathology, Anatomy and Physiology, University of Navarra School of Medicine, Pamplona, Spain

**Keywords:** co-design, competencies, curriculum development, delphi, learning objectives, undergraduate medical education

## Abstract

**Introduction:**

As Artificial Intelligence (AI) becomes embedded in clinical practice, medical graduates must be prepared to use it competently and critically. AI competencies and learning objectives (LOs) for medical students are needed. This study identifies these competencies and LOs for undergraduate medical education (UGME), particularly following the public release of large language models in 2022.

**Methods:**

A four-round modified Delphi process was conducted between May 2024 and January 2025. The panel included medical students (*n* = 14) and medical professors (*n* = 2) in the first three rounds, and international experts in medical education and AI in the final round (*n* = 10). In Round 1, using an open-ended question, 77 potential LOs were identified. In Rounds 2 and 3, these items were rated using a 7-point Likert scale (1 = “Low Priority”, 7 = “High Priority”), and participants were provided an area for comments. Median scores and interquartile ranges were used to assess the strength of agreement, and the LOs were modified accordingly. In Round 4, validation input was sought from 10 international experts in medical education and AI. Learning objectives were organized into categories representing broader competencies.

**Results:**

The process identified 7 competencies and 37 supporting LOs essential for medical graduates. Timelines for incorporating these across UGME were also identified as guidelines.

**Discussion:**

This study offers a framework for integrating AI into UGME, combining theoretical understanding with ethical use to prepare future physicians for clinical practice. Developed with input from students and multinational AI expert stakeholders, these competencies and LOs constitute a student-partnered, internationally informed framework that may guide curricular development in other settings.

## Introduction

Artificial Intelligence (AI) is fundamentally transforming healthcare and medical education (1–3). As a result, medical schools worldwide must prepare students to understand and use AI effectively, professionally, and ethically (4–8). There is a growing global imperative to define the AI competencies and the learning objectives (LOs) that undergraduate medical curricula should use to equip future physicians (9–12).

Given the rapid pace of emerging AI tools, there is an urgent need to establish evidence-based recommendations for how curricula should adapt (13–17) and the pedagogical methods for acquiring them. In addition, considerations regarding their international applicability should be based on global expert consensus (6, 18, 19).

Two prior Delphi studies, conducted among Korean and Turkish participants (20, 21), defined competencies for medical students. Additionally, a qualitative study in the United States (22) identified a set of competencies that healthcare professionals should acquire regarding AI. However, these studies predated the widespread release and availability of large language models (LLMs), and their significant impact on both the landscape of medical education and the practice of medicine starting in late 2022 (23–30).

We therefore aimed to define an updated set of AI-related competencies that students should develop during their undergraduate medical education (UGME), and the associated LOs that operationalize them. Furthermore, we identified potential timelines in the curricula where these competencies and LOs may be addressed. The present study is part of an initiative to integrate AI into the UGME curriculum at the University of Navarra. The effort employed a co-design approach, with students and professors collaborating to offer a post-LLM introduction, student-partnered, internationally informed framework with proposed curricular timing (31, 32).

## Methods

### Study context and student-faculty partnership

A co-design approach was chosen for this study as well as for the broader initiative (31, 32) because students are already early adopters of AI and important stakeholders in their education. Co-design is a collaborative process in which educators, students, and other stakeholders work together to design educational programs (33, 34). It shifts from a traditional, expert-driven curriculum development approach to a collective, inclusive one, aiming to create more relevant education that reflects real-world needs while incorporating diverse perspectives (35). In our case, it provided an opportunity to nurture scholarship among students, as it comprised four studies: a scoping review (36), a qualitative study of medical students (37), and two Delphi studies—one on the principles for the use of generative AI (38), and one defining AI competencies and LOs, which is the present report. At the start of the initial overall project (31), 200 first-year medical students in the 2023–2024 academic year were invited to participate, and 20 volunteered; 14 participated in this study. This group included advanced users, early adopters, and non-LLM users. All participants were involved in the overall design of the initiative, coached by experienced medical educators (JP, JS), and then divided into smaller groups to work alongside experienced researchers in the four associated studies.

Before undertaking the Delphi process, students and professors first participated in a process intentionally designed to educate the group about AI and medical education. These important steps were embedded into the research project to ensure the students were able to participate as panel experts for this study. This included presentations from AI experts and a journal club where the group discussed several key articles on AI in medical education. The aforementioned scoping review related to AI in UGME (36), a qualitative study (37), and a Delphi study (38), along with expert input, informed the present study.

The research team conducted a modified, four-round Delphi study involving a panel comprising medical students and medical educators from our university, international medical educators, and AI experts. Round 1 took place in May 2024, followed by three subsequent rounds conducted between September 2024 and January 2025. During this period, the LLMs in common public use included OpenAI's ChatGPT (GPT-4 and GPT-4o), Google Gemini (1.5 Pro/Flash), Anthropic Claude (Claude 3.5 Sonnet), and Meta Llama.

The format was modified to include not only AI experts and medical educators but also medical students, who represent an important group of early adopters, users, and stakeholders. As described by Niederberger et al., students can be considered “expert participants” (39). Our inclusion of medical students as panelists reflects contemporary Delphi methodology, in which the definition of “expert” extends beyond professional position or academic attainment to encompass those with specific lifeworld experience, including users of the systems under study (39).

This broadened conception is well established in consensus research across disciplines. For example, the multidisciplinary task force that was used in the EULAR recommendations study (40) included nurses, allied health professionals, and patient representatives alongside physicians. In studies involving the development of occupational health, stakeholder-engaged consensus methods were also utilized (41). Supported thus by the literature, we informed our students about AI and medical education and treated them as expert participants in the methodological sense defined above, and solely with respect to their standing as contributors who collaborated hand-in-hand with medical educators in Rounds 1–3 of the Delphi procedure.

### Study design and overview

A Delphi method was chosen because it uniquely gathers expert judgment and identifies agreement and disagreement. We used a conventional two-survey Delphi core with a qualitative generation round but included an additional fourth round as an external validation phase. The four-round structure was selected *a priori* deliberately: a qualitative idea-generation round (Round 1) followed by two structured rating rounds (Rounds 2 and 3) to test and stabilize consensus under predefined decision rules, and a final external validation round (Round 4) in which a separate, multinational expert panel appraised the competencies and LOs. The objective of each round is stated at the start of the corresponding subsections below. The study follows the DELPHISTAR recommendations (39) (Supplementary 1).

### Participants

The participants in the first three rounds of the Delphi process were 14 first-year medical students and two medical educators (JP, JS) at the University of Navarra. The group included students from the United States of America, Canada, Mexico, Poland, Switzerland, Austria, China, and Spain.

In the final round, the validation panel of experts included medical educators (*n* = 9) and an AI business expert (*n* = 1) from the United States of America, Canada, Chile, and Argentina. Expertise was defined to capture two complementary domains: (i) substantive expertise in AI applied to medicine and medical education, and (ii) expertise in medical-education curriculum design and governance. The overall panel was required to include both expertise domain profiles (21). In addition, panelists were eligible if they satisfied at least two of four criteria: (a) educational leadership and curriculum governance, (b) scholarship or formal training in health-professions education, (c) clinical practice with a teaching responsibility, and (d) demonstrated expertise in AI applied to medicine or health-professions education. Over 75% of the panel held both clinical and academic roles, and 7 had AI expertise (Supplementary 2). Experts were identified through the investigators' networks. The panel size of ten is consistent with Delphi methodology in which no unequivocal size standard exists (42). Published panels most commonly comprise 10 to 100 members, and small, purposively selected panels are considered acceptable when they adequately represent the relevant domains of expertise (42, 43). Therefore, the panel was deliberately composed to cover the two predefined complementary expertise domains.

### Ethics approval and consent to participate

Participation was voluntary, and informed consent was obtained from all participants. All responses were anonymous (44, 45). The primary researcher, however, was aware of the participants' identities to guarantee a high response rate, following Sumsion's recommendation that a Delphi study reach at least a 70% response rate in each round (44, 46). All data were de-identified prior to analysis and panel-wide feedback. The University of Navarra Research Ethics Committee reviewed and approved the study protocol (2024.084 mod1).

### Data collection procedures

To mitigate researcher influence, several safeguards addressed potential authority gradients between the professors and the first-year medical students. We intentionally practiced reflexivity in this study (47, 48), including disclosing structural power dynamics, team discussions in which the students were encouraged to think independently, and tracking all data responses. Participants prepared their eight candidate LOs independently before the in-person session, limiting real-time framing. All subsequent rating rounds used anonymized aggregated feedback, expressly to counter authority gradients and “groupthink” (44, 45). The consensus criteria were predefined *a priori* and applied unchanged. Every merger, rewording, and exclusion performed in the lead investigator's synthesis is documented, and the full framework was subsequently appraised by an independent, multinational expert panel in Round 4 (Supplementary 3–S6).

#### Round 1

The objective of Round 1 was to generate the initial set of learning objectives. We opted to follow a qualitative approach, using an open-ended question: “What are the AI LOs for undergraduate medical education (UGME)?” An in-person meeting was held with the 14 students and two professors (JS, JP) in May 2024. Each participant had been asked to bring eight candidate learning objectives. During the session, all proposed objectives were collected, and, in real time, identical or closely similar objectives were compiled and grouped. Each group was then thematically analyzed (49), and a single representative LO was rewritten to capture its theme. Following the session, the two lead professors (JS, JP) conducted a secondary thematic synthesis in June 2024, merging overlapping LOs by consensus, based on discussion and agreement, so that the items were ready for rating in Round 2.

#### Round 2

An online questionnaire using Google Forms was sent to participants in September 2024 (Supplementary 3) to subject the initial LOs to structured ratings. The questionnaire included the LOs from the previous round, and participants indicated their level of agreement or disagreement with each statement (LO) using a 7-point Likert scale (1 = “Low Priority”, 7 = “High Priority”). The median and interquartile range (IQR) were calculated for each statement. The anonymized results were shared with participants in an online forum, allowing them to provide additional feedback. The two lead researchers (JS, JP) then reviewed the feedback and incorporated suggestions to reword and/or merge LOs. Objectives that met the *a priori* consensus criteria (median ≥ 6 and IQR ≤ 2; see “Data analysis and consensus criteria”) were retained; those with a high but dispersed rating underwent a structured review of participants' comments, and the lowest-rated LOs were removed.

#### Round 3

A second online questionnaire with the updated LOs was distributed to participants in October 2024 to test whether consensus remained stable when the panel re-rated the LOs (Supplementary 4). A similar approach to that in Round 2 was used to provide input, undertake analyses, make modifications, and select the LOs for the next round. Applying the same consensus criteria from Round 2, together with a structured review of comments, the LOs were refined and organized into groups representing overarching competencies.

#### Round 4

In the final round, an online questionnaire with the remaining LOs and their overarching competencies was distributed from November 2024 to January 2025 to a separate multinational expert panel (Supplementary 5). This Round was designed *a priori* as an external validation and refinement phase rather than a further rating round. Hence, the questionnaire had a categorical response format of “Agree,” “Disagree,” or “Agree with changes” for each LO. In addition, qualitative data were collected via open-ended text boxes for optional comments on each LO and competency. The final step involved a meeting of the primary research team to refine, by consensus (which involved open discussion and voting), the final set of AI competencies and LOs, using input from the Round 4 external experts and determine their point of incorporation within UGME training.

### Data analysis and consensus criteria

For Rounds 2 and 3, the median and interquartile range (IQR) were calculated for every LO. Because ratings on the 7-point Likert scale are ordinal, the median was used as the primary measure of central tendency and consensus (44, 50), and the IQR as the paired measure of dispersion (51). No universally accepted consensus threshold exists in the Delphi literature (51, 52). Since the LOs were intended to govern conduct in medical training, an LO was retained when its median was ≥6 (i.e., “High Priority” or above on the Likert scale), and its IQR was ≤2. LOs with a median ≥ 6 but an IQR > 2 were regarded as showing high but dispersed agreement and underwent a structured qualitative review of participants' free-text comments, after which they were retained, reworded, consolidated with related LOs, or excluded. These criteria were defined *a priori* and applied unchanged in Rounds 2 and 3.

Participants' comments were analyzed thematically after each round. For items meeting the retention criteria, comments were screened for wording refinements. For items showing insufficient agreement, the structured qualitative review examined whether the dispersion reflected disagreement with the item's substance (leading to exclusion), its wording (leading to rewording), or its overlap with other items (leading to consolidation).

A summary of item flow across the four Delphi rounds is shown in Figure 1 and documented in the supplementary materials (Supplementary 6, Supplementary Table S1).

**Figure 1:**
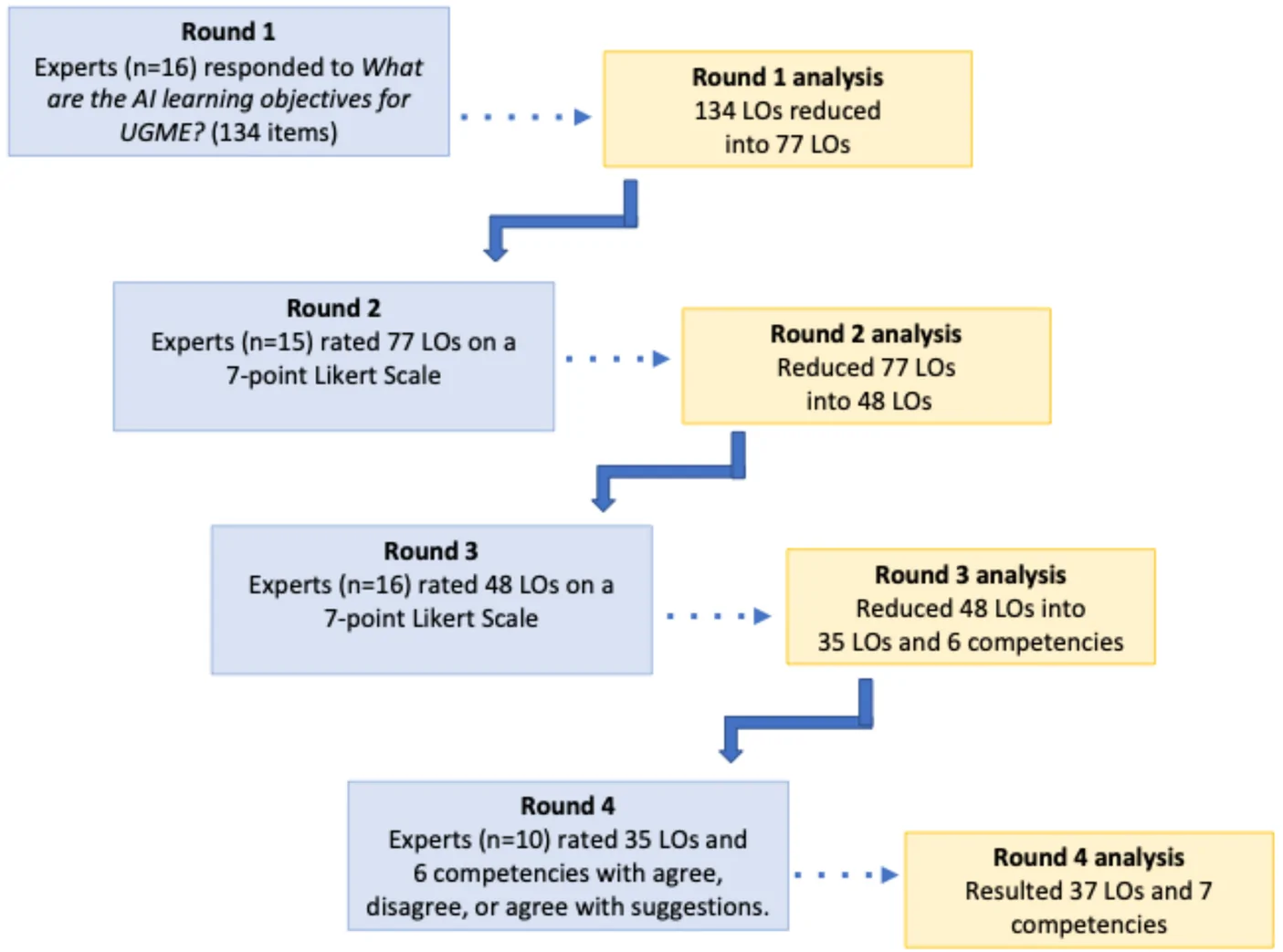
Delphi study overview: flow diagram of the evolution of the set of LOs across rounds (LOs = learning objectives)

## Results

In Round 1, all 16 panelists participated and generated 134 potential LOs. Real-time grouping during the in-person session, followed by the lead investigators' (JS, JP) secondary thematic synthesis, merged conceptually redundant items into a consolidated set of 77 LOs that formed the basis for Round 2 (Figure 1).

In Round 2, 15 of 16 participants provided feedback (94% response rate). Fifty-two of the 77 LOs had a median ≥ 6; of these, 46 had an IQR ≤ 2 and thus met the consensus criteria, while six had a high median but dispersed ratings (IQR > 2, one as high as 3.5) and were reviewed qualitatively. The remaining LOs had a median ≤ 5. After the consensus criteria were applied, along with a structured review of comments and after rewording, merging overlapping objectives, and removing the lowest rated, 48 LOs remained and advanced to Round 3 (Figure 1). The detailed ratings and disposition of the 77 candidate LOs (retained, merged, reworded, or excluded) are provided in the supplementary materials Supplementary 6, Supplementary Table S2.

The initial analysis of voting and comments from Round 3, with 16 of 16 participants providing feedback (100%), identified 42 LOs with a median ≥ 6, of which 41 also had an IQR ≤ 2; one had a median ≥ 6 but an IQR of 3 and was reviewed qualitatively. Six items received median scores of 4–5.5 (IQRs of 1–2), suggesting a stronger consensus that these items were less essential. After collating the input, incorporating suggestions, and merging and rewording LOs, the list was reduced to 35 LOs, grouped into six overarching competencies (Figure 1). The complete details can be found in the supplementary materials (Supplementary 6, Supplementary Table S3).

Experts (*n* = 10) in Round 4 agreed that all 35 LOs and six competencies were appropriate; agreement was unanimous. However, rewording was suggested for two competencies, and one new competency was proposed: “critically appraise the potential strengths, limitations, risks and bias of AI in medical education and healthcare”. Among the 35 LOs, 20 were accepted by the experts without changes, 15 received suggestions for rewording or merging, and four new LOs were proposed (Figure 1). A final step involved a meeting of the primary research team to refine, by consensus (which involved open discussion and voting), the final set of AI competencies and LOs. Items newly proposed by the external experts in this Round 4 (one competency and four LOs) were integrated through consensus at this final step, prompting the merging of two existing LOs due to content overlap (Supplementary 6, Supplementary Table S4). The net effect was an increment of two LOs (four newly proposed LOs were added to the existing 35 validated LOs, two of which were merged, resulting in 37 final LOs). The newly introduced competency and the two net-new LOs are marked with asterisks in Table 1. As they originated from the validating panel itself and were refined, rather than contradicted, the consensus stabilized in Rounds 2–3; no further rating rounds were undertaken. This process resulted in a final set of seven competencies and 37 LOs (Table 1, left & mid columns).

**Table 1:** Final framework (seven competencies, 37 learning objectives) and recommended year of study.

#	Final competency/learning objective	Year to address
1.	Define and describe AI.	
1.1	Define what AI is	First
1.2	Describe the basic principles of how AI systems are “trained” (e.g., supervised, unsupervised, and reinforcement learning) and provide examples of their application in healthcare, such as diagnostic tools or treatment recommendations.	First
1.3	Explain the concepts related to AI, such as traditional AI, machine learning (ML), deep learning (DL), natural language processing (NLP), large language models (LLMs), generative AI, data-driven algorithms, augmented reality, and virtual reality.	First
2.	Use AI effectively, responsibly, and professionally to support learning in medicine.	
2.1	Analyze the ways AI can support or hinder learning in medical education, with examples of benefits (e.g., tailored learning tools) and risks (e.g., overreliance or misinformation).	First
2.2	Create effective prompts to support supervised educational activities including learning about clinical decision-making and patient education.†	First
2.3	Recognize errors and biases within AI-derived information.	First
2.4	Recognize and select the most appropriate AI technology for the specific task or need during learning.	First
3.	Critically appraise the potential strengths, limitations, risks and bias of AI in medical education and healthcare.*	
3.1	Describe how AI could enhance clinical reasoning (e.g., by providing data-driven insights) or undermine it (e.g., through errors or biases) with examples in healthcare scenarios.	Pre-clinical
3.2	Apply critical thinking when analyzing AI-generated information in medical learning.	First
3.3	Apply strategies to mitigate the risks of overreliance on AI in clinical reasoning and medical learning.*	First
3.4	Explain the strengths and weaknesses of AI technologies used in medical education.	First
3.5	Identify potential opportunities and risks associated with AI technologies and propose strategies to address them.	Pre-clinical
3.6	Implement strategies to mitigate risks associated with AI use in medical education, such as ensuring data privacy, avoiding overreliance on AI tools, and maintaining academic integrity.	First
4.	Explain the role of AI in supporting clinical care, research and quality improvement.	
4.1	Provide examples of the applications of AI in the various fields of medicine (e.g., clinical diagnostics and decision-making, health records management, screening, education, and research).	Clinical
4.2	Describe strategies to avoid risks or pitfalls related to using AI in clinical practice.	Clinical
4.3	Describe the use of AI to support clinical workflows, documentation and scheduling.	Final
4.4	Describe the application of AI technologies in clinical settings, including their roles in diagnostics, treatment planning, patient monitoring, and risk assessment.	Final
4.5	Apply critical thinking including assessing the accuracy when analyzing AI-generated information in clinical practice.	Clinical/Final
4.6	Respond to patients in a way that demonstrates collaborative, shared decision-making and best evidence-driven care when patients approach with information they found using AI.	Clinical/Final
4.7	Provide examples of how AI can assist in medical research.	Clinical
4.8	Use AI tools effectively and appropriately to assist with literature searches, evaluate the relevance of findings, and critically appraise the quality of research articles.	Pre-clinical
4.9	Recognize the limitations and biases of AI tools in medical research and their potential impact on literature searches and data analysis.	Pre-clinical
4.10	Provide examples of how AI can support quality improvement, including using data in health records to identify opportunities for improvement.	Clinical/Final
4.11	Describe the role of large datasets and AI analytics in supporting quality improvement and advancing research in healthcare.	Final
4.12	Explain the strengths and weaknesses of AI technologies used in healthcare.	Clinical
5.	Identify and apply best ethical and legal practices when using AI to support learning and clinical care.	
5.1	Describe the ethical considerations when using AI for learning, medical practice, health records keeping and analyses, research, and quality improvement.	First—Clinical
5.2	Describe ethical risks and or pitfalls related to using AI in clinical practice.	Clinical
5.3	Explain the legal implications of AI use in healthcare, including potential risks and methods to mitigate these.	Pre-clinical
5.4	Describe best practices and regulatory requirements for ensuring data protection, privacy, anonymity, and informed consent when using AI in healthcare.	Clinical/Final
5.5	Describe ethical considerations of using large datasets in healthcare, such as privacy, security, and data-sharing challenges.*	Clinical
5.6	Practice professionalism by acknowledging the use of AI when using technology in medical education, scholarship, and submitting assignments.	First
5.7	Practice in a person-centered, humanistic way (empathy, compassion, person-centeredness) when using AI and other technologies in clinical care.	Clinical/Final
5.8	Be transparent with patients and care providers when AI used in clinical care (e.g., making diagnostic or treatment decisions).	Clinical/Final
6.	Value the role of interdisciplinary teamwork in AI-related initiatives and projects.	
6.1	Explain the importance of a multidisciplinary team approach to AI-driven healthcare projects, clearly defining the physician's role and the communication skills required for effective collaboration.	Clinical/Final
6.2	Apply a multidisciplinary approach when adopting or using AI in clinical practice, collaborating effectively with data scientists, engineers, and other experts to ensure patient-centered, evidence-based solutions.	Clinical/Final
7.	Demonstrate curiosity and awareness of emerging AI technologies, trends and applications.	
7.1	Undertake continuous learning to remain updated on emerging AI technologies and practices in healthcare.^†^	Final
7.2	Adapt to new AI technologies and methodologies as they emerge, while critically assessing their relevance and impact on clinical practice and patient outcomes.	Final

* One Competency and two LOs were proposed by the Round 4 external expert panel.

† Clearer wording was proposed during peer review.

Regarding the recommended year in which each LO should be addressed within the curriculum, this was not itself an outcome of the Delphi process. Rather, these recommendations were developed by the research team through our own analysis of the Delphi findings and are offered as curricular guidance (Table 1, right column).

## Discussion

This study defines seven AI competencies and 37 supporting LOs that undergraduate medical students should acquire during their education. These competencies and LOs were determined through a rigorous process, which involved updating participants on current AI literature, integrating evidence from a scoping review of AI literature related to UGME up to April 2024 (36), undertaking a qualitative study of medical students’ use of AI (37), and conducting a modified Delphi process to determine AI principles of use for medical students (38), with participants who represent stakeholders in the area (31, 32).

Notably, the competencies and LOs incorporate LLMs. Prior studies identifying AI-related competencies for UGME were published before the launch of ChatGPT, one of the most widely used LLMs, in November 2022. The specific contribution of the present study is therefore best framed as a post-LLM introduction, student-partnered framework with proposed curricular timing, updating pre-LLM competency studies whose participants may not have anticipated current LLM capabilities. An important aspect of this study's uniqueness lies in its co-design approach (34): the medical students involved were not merely research participants but co-designers and co-authors, engaged as peer experts alongside their supervising professors. Moreover, these students began their medical training at the very moment LLMs became generally available. They thus belong to the first cohort of medical students to be early adopters of AI and LLMs from the outset of their education, giving them both first-hand insight into these tools and a vested interest in how future physicians are prepared to meet emerging needs (32).

In interpreting the framework, it is important to keep in mind that competencies and LOs occupy distinct conceptual levels. The seven competencies are overarching capabilities (enduring habits and abilities that graduates must ultimately possess), whereas the LOs beneath each competency are the concrete, content-acquisition or isolated-skill execution through which that capability is progressively built. Where similar wording appears at both levels, their function differs: the competency denotes what must be achieved, and the LO denotes the goals through which it is achieved. Likewise, the recurrence of themes such as bias, risk, and critical appraisal across competencies is deliberate rather than redundant. The same critical skill is exercised in different contexts, such as learning (Competencies 2- 3), clinical care (Competency 4), and ethical-legal practice (Competency 5), and at different curricular stages. For example, LO 3.4 and LO 4.12 apply the same appraisal skill to educational and healthcare technologies and are addressed in different curricular years; LO 3.2 and LO 4.5 apply critical analysis of AI output in learning and in clinical practice, where an explicit accuracy-assessment expectation is added; and LO 2.3 and LO 4.9 address recognition of errors and bias in general use and in research and literature work.

The affective formulations of competency level are likewise deliberate: “valuing” is a defined level of the affective domain of Bloom's taxonomy and is assessed through the demonstrable LOs beneath it (6.1, 6.2). The AI-PACE framework for integrating AI into medical education recommends establishing LOs across the cognitive, psychomotor, and affective domains (53).

Each LO should likewise be read within the overarching competency in which it resides. LO 2.2, for example, sits under Competency 2 (Use AI effectively, responsibly, and professionally to support learning) and therefore concerns acquiring prompting skills in supervised educational contexts, but it does not propose that early-stage students employ AI for clinically consequential decisions or patient-facing guidance. LOs involving genuine clinical application are located under Competency 4 and are recommended for the pre-clinical, clinical and final years, while first-year LOs are deliberately confined to foundational AI literacy, supervised educational use, and critical appraisal of AI outputs (e.g., 2.3, 3.2, 3.3, 3.6).

Three prior studies, Lee et al., Caliskan et al., and Russell et al., defined AI-related competencies (20–22). The first two focused on competencies for medical students, while the third addressed healthcare professionals more broadly. Lee et al. (20) and Caliskan et al. (21) employed Delphi studies, whereas Russell et al. (22) utilized a qualitative design followed by a consensus process involving the research team. Because those studies were conducted before the release of ChatGPT, our findings reflect a more current AI landscape. In addition, while prior studies were limited to a single country, our work includes participants from multiple countries.

Lee et al. developed essential and optional AI competencies, distinguished through two separate, two-round professional expert Delphi panels and a nationwide survey among Korean students and professors (20). However, the study did not include students in either Delphi panel. In Caliskan et al., the expert group consisted predominantly of individuals with advanced education levels in Turkey, including 40% who held academic titles, with three medical students among the 75 experts (4%) (21). Russell et al. conducted research in the United States without any student input (22).

Our approach uniquely engaged students and faculty, working together from the outset, with a fourth round added to incorporate external international expert confirmation. In our experience, the students approached the process with open, inquisitive minds, demonstrating a high level of commitment to ensure their training was optimal. Their fresh, formative-stage perspective enriched the research process. In addition, given that today's medical students are digital natives (54) who routinely engage with AI tools (55), we believe they are distinctively positioned to critically reflect and provide input regarding the role of AI in medical education (56–59). While they may not possess deep technical expertise, their lived experience and familiarity with these technologies, particularly when supplemented by formal education in AI literacy (60–64), equip them with valuable insights into AI's practical applications and implications in clinical and educational settings (32, 37). Lastly, the literature supports the notion that students’ opinions should be taken into consideration regarding how AI is integrated into their medical education (65). In summary, first-year students were central to the generative rounds for four reasons: (i) as co-designers, they are legitimate stakeholders in the curriculum that will shape their training; (ii) as digital natives, they are documented early adopters of LLMs; (iii) they entered the process only after structured preparation (expert presentations, a journal club, and a scoping review on AI); and (iv) contemporary Delphi methodology recognizes such informed users as expert participants (39). The resulting framework nonetheless remained subject to external validation by the multinational expert panel in Round 4.

Regarding the competencies and LOs, there is strong consistency with previous findings (20–22), underscoring foundational knowledge of AI definitions and core concepts, as well as the need to understand essential skills, including the ethical, legal, and societal implications of AI use in medicine. The application of AI in clinical settings (supporting clinical diagnosis, AI-enhanced encounters, practical applications in treatment planning, and clinical decision-making), as well as its critical appraisal (evaluating data, evidence, strengths, limitations, and biases of AI tools), is another common area of concurrence. Data management and analysis were also found to be crucial for handling, processing, analyzing, and evaluating medical data (20). Interdisciplinary teamwork was highlighted in our study as well as in two studies (21, 22), underscoring the importance of collaboration across disciplines and acknowledging the involvement of medical professionals, data scientists, and engineers in optimizing AI applications in healthcare. Our findings align with two studies (21, 22) that also emphasize the importance of adapting to new AI technologies and staying current with emerging AI trends for medical practice. Lastly, frameworks published after our study was first designed reinforce this, including a medical-AI competency framework integrating the UNESCO AI competencies with the Miller pyramid (66) and an implementation-oriented model for embedding AI competence at the licensure level (67). In contrast, while Caliskan et al. found weak consensus on several foundational AI competencies (such as how AI systems are trained, understanding limitations, strengths, and weaknesses, and communicating AI use to patients) (21), our panel reached strong consensus for these LOs as well as for LOs related to explaining AI's role in supporting clinical care and research. Similarly, whereas Lee et al. suggest that competencies related to the application of AI in clinical practice were mainly optional (20), our results identify them as indispensable. This discrepancy may be attributable to the rapid and ongoing advancements in AI.

In addition, possibly due to publicly available LLMs, new competencies and LOs surfaced from the previous studies, including a greater emphasis on learning, such as the use of AI to support education. These included creating effective prompts when using LLMs, critically evaluating AI-generated information (including literature searches), and ensuring academic integrity when using LLMs.

Importantly, we identified LOs related to the clinical setting, including patient interactions, patient-centered practices, and empathy. Our competencies and LOs also emphasize transparency in clinical decisions involving AI and shared decision-making. Finally, the importance of utilizing AI for quality improvement, managing records, streamlining clinical workflows, and enhancing clinical documentation tasks was newly outlined.

Positioning the present framework against this rapidly growing literature clarifies what is confirmatory and what is novel. Confirmatory elements include foundational AI knowledge, ethical and legal practice, critical appraisal, interdisciplinary teamwork, and adaptability, which recur across recent curriculum frameworks and scoping reviews of AI education programs (68, 69). Novel elements include the post-LLM introduction, learning-focused objectives (prompting, academic integrity, AI-assisted literature work), the patient-facing and shared decision-making objectives, the proposed curricular timing, and the student-partnered development process itself (69, 70). To facilitate integration into the existing competency-based curricula, the seven AI competencies defined here were mapped to CanMEDS roles and ACGME core competencies (Supplementary 7).

Following our data collection (Jan 2025), the literature reveals three new emerging themes: contingency planning for when AI is unavailable or unreliable (70, 71), regulation of confidence and uncertainty (72), and metacognitive effects of AI-supported reasoning (73). None of these, to our knowledge, have been explicitly stated as assessable learning outcomes. Recent consensus studies without students show the same pattern, including an international Delphi of 211 experts from 79 countries (74), a faculty-led Canadian Delphi (75), a Chinese expert consensus (76) and a patient-partnered consensus (77). Monteith et al. highlight the risk of students becoming over-reliant on AI, thereby reducing the quality of clinical reasoning and decision-making, negatively impacting patient communication, and raising the potential for deskilling (78), while Ke et al. propose a competency-protective framework for learning in the era of AI (79). Our framework specifically addresses these risks; LOs 2.1, 3.1, 3.3 and 3.6, for example, target overreliance and the preservation of AI-independent reasoning. Finally, a recent scoping review of proposed AI competencies for undergraduate medical education, which synthesizes 564 competency statements from 54 studies across 22 countries, identified seven domains that map directly onto our seven competencies (80).

Viewed through a digital health lens, the framework addresses the user-preparedness layer of safe AI implementation, training the human side of the future human-AI system before graduates encounter clinical AI. Several LOs directly target known human-factor risks: automation bias and cognitive overreliance (2.1, 2.3, 3.3, 3.6) (71, 81); human-AI interaction and user preparedness (Competencies 2–3); the safe integration of AI into clinical workflow and documentation (4.3, 4.4); accountability and transparency in AI-assisted care (5.6, 5.8); and patient-facing AI use with shared decision-making (e.g., 4.6, 5.7). In this sense, the framework speaks not only to curriculum design but to safe implementation of digital health technologies.

Our framework recommends the integration of AI literacy through a flexible, horizontal approach that addresses it longitudinally across the entire UGME curriculum (4, 31). Although these LOs may seem like a large number to add to an already packed curriculum, many of them can be incorporated into existing classes (82). Foundational LOs should be introduced early in the curriculum, ideally in the first year, to provide a baseline understanding of AI principles and their relevance to medicine (83, 84). Subsequent LOs can then be embedded within current courses across the basic, preclinical, clinical, and medical humanities classes (9, 75, 82, 85). This scaffolded integration aligns with best practices in curriculum design, where early exposure followed by progressive reinforcement has been shown to enhance retention, contextual application, and professional identity formation (86, 87). Embedding AI literacy in this manner also ensures that learners appreciate both the technical foundations and the ethical, clinical, and societal implications of AI as they advance through their medical education (87, 88).

Institutions with differing levels of AI infrastructure can adapt this scaffold. Foundational LOs can be delivered with freely accessible, general-purpose LLMs and open-access educational resources. In addition, adoption can proceed in phases, prioritizing foundational literacy in areas with limited infrastructure (69, 82).

### Limitations

This study has several limitations. First, the panel was small, with 14 students and two medical educators in Rounds 1–3, and ten external experts in Round 4, although these sizes are consistent with Delphi methodology norms (42, 43). Second, the first three rounds were conducted within a single medical school; although participants represented multiple nationalities, their educational experiences largely derived from a single institutional context. However, the professors and external experts brought experiences from other medical schools. Third, students outnumbered other panelists in the generative and rating rounds. Although their input reflects the perspectives of informed users, given the orientation process they participated in prior to the study, their input may embody perceived preferences shaped by limited clinical exposure. Fourth, some researchers' influence cannot be fully excluded; the lead investigators performed the thematic synthesis. Fifth, despite intentional mitigation strategies, including open discussions and reflexivity, the professor-learner power differential may have influenced the students' input. Sixth, the study did not include patients or members of the public, whose perspectives on the use of AI in medical education, particularly regarding patient-facing use, transparency, and shared decision-making, were not considered. Seventh, the framework has not been evaluated. No implementation, feasibility, assessment-validity, or outcome data are available, and further steps, such as those outlined in the Conclusions, are required before any effect on learning, competence, or practice can be claimed. Finally, a consensus process reflects the expertise represented within its panel, and the agreement reported here should be understood as establishing the priorities this panel recognized rather than as establishing the completeness of the curriculum.

## Conclusions

Since ChatGPT and other LLMs became publicly available for medical students, the need to provide them with a deep, formal, and professional education in the use of AI during their undergraduate education has become urgent. Through a systematic consensus procedure within a student-faculty panel, externally validated by a multinational panel of medical education and AI experts, we propose a student-partnered, internationally informed framework that defines what medical students should learn about AI, and when. This study, however, does not demonstrate that acquiring these competencies will improve AI literacy, clinical reasoning, ethical decision-making, patient safety, or clinical outcomes. To achieve this, necessary next steps would be implementation studies and evaluation, for example, pre/post competence assessment and longitudinal follow-up. Finally, this framework should be treated as a living document, given the pace of AI development. Institutions should revisit it on a defined cycle within routine curricular review, which is a need the framework itself anticipates through Competency 7.

## Data Availability

The original contributions presented in the study are included in the article/Supplementary Material, further inquiries can be directed to the corresponding author/s.
