# Supplementary material for: What and when: a Delphi study identifying AI competencies and learning objectives for medical students
Source: Front Digit Health. 2026 Sep 30;8:1918847. doi: 10.3389/fdgth.2026.1918847 (PMC13662149; doi:10.3389/fdgth.2026.1918847)
Supplement: Supplementary file 1 [file Supplementaryfile3.pdf]

AI learning objectives Round 2

Survey goal:
To rate the importance of the following learning objectives for you to achieve upon graduation. Please indicate how essential each learning objective is for you, considering the knowledge, attitudes and skills you will have obtained.

Please read through the attached document first and then set aside 20-30 minutes to answer the questions.

AI learning objectives table

Please note that the objectives have been arranged in temporary categories. We will later collectively confirm the final categories as well as competencies.

1. Email *

____

Skip to question 12

Foundational information

Using a scale of 1 to 7, where 1 indicates lowest priority and 7 represents highest priority, assign a priority for each objective listed below.

2. Describe the historical evolution of AI

Mark only one oval.

	1	2	3	4	5	6	7	
Low	◯	◯	◯	◯	◯	◯	◯	Highest priority

3. Describe how AI systems are “trained” (e.g. supervised, unsupervised and reinforcement learning).

Mark only one oval.

	1	2	3	4	5	6	7	
Low	◯	◯	◯	◯	◯	◯	◯	Highest priority

4. Define what AI is.

Mark only one oval.

	1	2	3	4	5	6	7	
Low	◯	◯	◯	◯	◯	◯	◯	Highest priority

5. Define, and differentiate between, concepts related to AI such as large language modeling (LLM), machine learning (ML), deep learning (DL), traditional AI, and generative AI.

Mark only one oval.

	1	2	3	4	5	6	7	
Low	◯	◯	◯	◯	◯	◯	◯	Highest priority

6. Name the different types of AI technologies, specifically as they relate to medicine (e.g. large language model agents)

Mark only one oval.

	1	2	3	4	5	6	7	
Low	◯	◯	◯	◯	◯	◯	◯	Highest priority

7. Differentiate AI from technologies such as augmented reality (AR) and virtual reality (VR), specifically concerning their use in health care.

Mark only one oval.

	1	2	3	4	5	6	7	
Low	◯	◯	◯	◯	◯	◯	◯	Highest priority

8. Summarize how AI algorithms work

Mark only one oval.

	1	2	3	4	5	6	7	
Low	◯	◯	◯	◯	◯	◯	◯	Highest priority

9. Select the most appropriate AI technology for the specific task or need at hand.

Mark only one oval.

	1	2	3	4	5	6	7	
Low	◯	◯	◯	◯	◯	◯	◯	Highest priority

10. Provide examples of the applications of AI in the various fields of medicine (e.g. clinical diagnostics and decision-making, health records management, screening, education, research).

Mark only one oval.

	1	2	3	4	5	6	7	
Low	◯	◯	◯	◯	◯	◯	◯	Highest priority

11. Create effective prompts for ChatGPT or other AI platforms

Mark only one oval.

	1	2	3	4	5	6	7	
Low	◯	◯	◯	◯	◯	◯	◯	Highest priority

AI in learning

Using a scale of 1 to 7, where 1 indicates lowest priority and 7 represents highest priority, assign a priority for each objective listed below.

12. Describe how AI can support, and undermine, learning (as a medical student), including in areas such as critical thinking.

Mark only one oval.

	1	2	3	4	5	6	7	
Low	◯	◯	◯	◯	◯	◯	◯	Highest priority

13. Use AI effectively, professionally, and ethically in support of medical learning in UGME. (Under graduate medical education)

Mark only one oval.

	1	2	3	4	5	6	7	
Low	◯	◯	◯	◯	◯	◯	◯	Highest priority

14. Use AI tools to enhance medical learning in a way that is effective and professional.

Mark only one oval.

	1	2	3	4	5	6	7	
Low	◯	◯	◯	◯	◯	◯	◯	Highest priority

15. Use effective prompts when using generative AI for learning.

Mark only one oval.

	1	2	3	4	5	6	7	
Low	◯	◯	◯	◯	◯	◯	◯	Highest priority

16. Identify the limits of AI in support of learning (when should we use it and when should we not use it

Mark only one oval.

	1	2	3	4	5	6	7	
Low	◯	◯	◯	◯	◯	◯	◯	Highest priority

17. Use AI to support learning with simulated patients

Mark only one oval.

	1	2	3	4	5	6	7	
Low	◯	◯	◯	◯	◯	◯	◯	Highest priority

18. Use generative AI effectively and professionally in case-based and problem-based learning.

Mark only one oval.

	1	2	3	4	5	6	7	
Low	◯	◯	◯	◯	◯	◯	◯	Highest priority

Clinical applications of AI

Using a scale of 1 to 7, where 1 indicates lowest priority and 7 represents highest priority, assign a priority for each objective listed below.

19. Evaluate clinical applications of AI

Mark only one oval.

	1	2	3	4	5	6	7	
Low	◯	◯	◯	◯	◯	◯	◯	Highest priority

20. Describe the strengths and limitations of AI systems in supporting clinical care, including in such as areas as critical thinking, and generating diagnoses and care plans.

Mark only one oval.

	1	2	3	4	5	6	7	
Low	◯	◯	◯	◯	◯	◯	◯	Highest priority

21. Describe ethical risks or pitfalls related to the use of AI in clinical practice.

Mark only one oval.

	1	2	3	4	5	6	7	
Low	◯	◯	◯	◯	◯	◯	◯	Highest priority

22. Implement strategies to avoid risks and pitfalls related to the use of AI in clinical practice.

Mark only one oval.

	1	2	3	4	5	6	7	
Low	◯	◯	◯	◯	◯	◯	◯	Highest priority

23. Evaluate the strengths and limitations of AI systems in clinical settings

Mark only one oval.

	1	2	3	4	5	6	7	
Low	◯	◯	◯	◯	◯	◯	◯	Highest priority

24. Interpret AI-generated outputs and integrate them into clinical decision-making

Mark only one oval.

	1	2	3	4	5	6	7	
Low	◯	◯	◯	◯	◯	◯	◯	Highest priority

25. Collaborate effectively with AI systems in clinical workflows

Mark only one oval.

	1	2	3	4	5	6	7	
Low	◯	◯	◯	◯	◯	◯	◯	Highest priority

26. Describe the use of AI technologies in support of clinical workflows (such as documenting patient visits in electronic health records optimizing clinical workflow ects.)

Mark only one oval.

	1	2	3	4	5	6	7	
Low	◯	◯	◯	◯	◯	◯	◯	Highest priority

27. Provide input for AI model development and validation

Mark only one oval.

	1	2	3	4	5	6	7	
Low	◯	◯	◯	◯	◯	◯	◯	Highest priority

28. Describe the process of providing input for AI model development and validation

Mark only one oval.

	1	2	3	4	5	6	7	
Low	◯	◯	◯	◯	◯	◯	◯	Highest priority

29. Adapt to changes in practice workflows due to AI integration

Mark only one oval.

	1	2	3	4	5	6	7	
Low	◯	◯	◯	◯	◯	◯	◯	Highest priority

30. Describe the application of AI technologies in clinical settings, including their roles in diagnostics, treatment planning and recommendations, patient monitoring, and risk assessment.

Mark only one oval.

	1	2	3	4	5	6	7	
Low	◯	◯	◯	◯	◯	◯	◯	Highest priority

31. Describe how AI could enhance or undermine clinical reasoning

Mark only one oval.

	1	2	3	4	5	6	7	
Low	◯	◯	◯	◯	◯	◯	◯	Highest priority

AI application in medical research

Using a scale of 1 to 7, where 1 indicates lowest priority and 7 represents highest priority, assign a priority for each objective listed below.

32. Describe the effective and ethical use of AI in medical research

Mark only one oval.

	1	2	3	4	5	6	7	
Low	◯	◯	◯	◯	◯	◯	◯	Highest priority

33. Describe ethical challenges and limitations posed by AI in medical research and strategies to mitigate these.

Mark only one oval.

	1	2	3	4	5	6	7	
Low	◯	◯	◯	◯	◯	◯	◯	Highest priority

34. Identify the opportunities and threats of the use of AI in medical research

Mark only one oval.

	1	2	3	4	5	6	7	
Low	◯	◯	◯	◯	◯	◯	◯	Highest priority

35. Use AI to enhance the critical appraisal of literature (studies)

Mark only one oval.

	1	2	3	4	5	6	7	
Low	◯	◯	◯	◯	◯	◯	◯	Highest priority

36. Evaluate the quality (including validity, reliability, sensitivity, specificity, generalizability, and applicability) of studies on the use of AI in medicine

Mark only one oval.

	1	2	3	4	5	6	7	
Low	◯	◯	◯	◯	◯	◯	◯	Highest priority

37. Critically analyze AI algorithms and models for clinical relevance and generalizability

Mark only one oval.

	1	2	3	4	5	6	7	
Low	◯	◯	◯	◯	◯	◯	◯	Highest priority

Use and apply

Using a scale of 1 to 7, where 1 indicates lowest priority and 7 represents highest priority, assign a priority for each objective listed below.

38. Create effective prompts for ChatGPT or other AI platforms

Mark only one oval.

	1	2	3	4	5	6	7	
Low	◯	◯	◯	◯	◯	◯	◯	Highest priority

AI applications in health records, data and practice management

Using a scale of 1 to 7, where 1 indicates lowest priority and 7 represents highest priority, assign a priority for each objective listed below.

39. Explain how AI can help with operational efficiency in administrative tasks such as scheduling patients, maintaining medical records, searching medical records, and writing medical notes

Mark only one oval.

	1	2	3	4	5	6	7	
Low	◯	◯	◯	◯	◯	◯	◯	Highest priority

40. Use AI technology in support of medical record keeping and retrieval of medical records.

Mark only one oval.

	1	2	3	4	5	6	7	
Low	◯	◯	◯	◯	◯	◯	◯	Highest priority

41. Use AI technology to support quality improvement using electronic health records.

Mark only one oval.

	1	2	3	4	5	6	7	
Low	◯	◯	◯	◯	◯	◯	◯	Highest priority

Critical reasoning

Using a scale of 1 to 7, where 1 indicates lowest priority and 7 represents highest priority, assign a priority for each objective listed below.

42. Learn how to apply critical thinking when using and applying AI output in practice

Mark only one oval.

	1	2	3	4	5	6	7	
Low	◯	◯	◯	◯	◯	◯	◯	Highest priority

43. Analyse information and data obtained by AI in healthcare

Mark only one oval.

	1	2	3	4	5	6	7	
Low	◯	◯	◯	◯	◯	◯	◯	Highest priority

44. Use information derived from AI in combination with other reliable sources of medical information to aid in clinical care and learning.

Mark only one oval.

	1	2	3	4	5	6	7	
Low	◯	◯	◯	◯	◯	◯	◯	Highest priority

Teamwork and interdisciplinary collaboration

Using a scale of 1 to 7, where 1 indicates lowest priority and 7 represents highest priority, assign a priority for each objective listed below.

45. Collaborate with data scientists, engineers, and other AI experts in healthcare settings

Mark only one oval.

	1	2	3	4	5	6	7	
Low	◯	◯	◯	◯	◯	◯	◯	Highest priority

46. Apply a multidisciplinary approach when adopting or using AI in clinical practice, including collaborating with data scientists, computer engineers, biomedical engineers, and other experts

Mark only one oval.

	1	2	3	4	5	6	7	
Low	◯	◯	◯	◯	◯	◯	◯	Highest priority

47. Participate in, and contribute medical expertise to, interdisciplinary AI projects related to the delivery of health care and health-related education.

Mark only one oval.

	1	2	3	4	5	6	7	
Low	◯	◯	◯	◯	◯	◯	◯	Highest priority

48. Foster teamwork, including effective communication across disciplines, when designing and using AI

Mark only one oval.

	1	2	3	4	5	6	7	
Low	◯	◯	◯	◯	◯	◯	◯	Highest priority

Communication (about AI) *("Human aspects")*

Using a scale of 1 to 7, where 1 indicates lowest priority and 7 represents highest priority, assign a priority for each objective listed below.

49. Explain AI-generated or enhanced processes and results to patients in a way that aligns with best communication practices

Mark only one oval.

	1	2	3	4	5	6	7	
Low	◯	◯	◯	◯	◯	◯	◯	Highest priority

50. Respond to patients in a way that demonstrates collaborative, shared decision-making and best evidence-driven care when they approach you with information they found using AI.

Mark only one oval.

	1	2	3	4	5	6	7	
Low	◯	◯	◯	◯	◯	◯	◯	Highest priority

Legal implications and considerations

Using a scale of 1 to 7, where 1 indicates lowest priority and 7 represents highest priority, assign a priority for each objective listed below.

51. Describe data protection and privacy laws in a specific jurisdiction when using AI in clinical practice

Mark only one oval.

	1	2	3	4	5	6	7	
Low	◯	◯	◯	◯	◯	◯	◯	Highest priority

52. Describe the regulatory landscape governing the development and use of AI technologies in healthcare

Mark only one oval.

	1	2	3	4	5	6	7	
Low	◯	◯	◯	◯	◯	◯	◯	Highest priority

53. Explain the legal implications of AI usage, including potential risks.

Mark only one oval.

	1	2	3	4	5	6	7	
Low	◯	◯	◯	◯	◯	◯	◯	Highest priority

54. Describe regulatory guidelines governing AI in healthcare (HIPPA, GDPR)

Mark only one oval.

	1	2	3	4	5	6	7	
Low	◯	◯	◯	◯	◯	◯	◯	Highest priority

Ethical and Professional use of AI

Using a scale of 1 to 7, where 1 indicates lowest priority and 7 represents highest priority, assign a priority for each objective listed below.

55. Identify ethical concerns when using AI technologies for learning, medical practice, health records keeping and analyses, research, and quality improvement.

Mark only one oval.

	1	2	3	4	5	6	7	
Low	◯	◯	◯	◯	◯	◯	◯	Highest priority

56. Practice ethically and professionally using AI technologies to support learning in medical education, clinical care, administration, research, and quality improvement.

Mark only one oval.

	1	2	3	4	5	6	7	
Low	◯	◯	◯	◯	◯	◯	◯	Highest priority

57. Describe issues of privacy, anonymity, consent, bias, transparency, and accountability with respect to the use of AI in medical practice and research.

Mark only one oval.

	1	2	3	4	5	6	7	
Low	◯	◯	◯	◯	◯	◯	◯	Highest priority

58. Apply best ethical and professional practices (e.g. ethical frameworks) to AI-powered decision-making processes and tools.

Mark only one oval.

	1	2	3	4	5	6	7	
Low	◯	◯	◯	◯	◯	◯	◯	Highest priority

59. Practice transparency and professionalism by acknowledging the use of AI generated sources of information in medical education, research, and clinical practice.

Mark only one oval.

	1	2	3	4	5	6	7	
Low	◯	◯	◯	◯	◯	◯	◯	Highest priority

60. Understand the ethical limits and considerations when employing AI

Mark only one oval.

	1	2	3	4	5	6	7	
Low	◯	◯	◯	◯	◯	◯	◯	Highest priority

61. Discuss the ethical implications of using AI (consequences)

Mark only one oval.

	1	2	3	4	5	6	7	
Low	◯	◯	◯	◯	◯	◯	◯	Highest priority

62. Apply the use of AI technologies responsibly and effectively when they are being used to aid in clinical care, including analysing results and decision-making

Mark only one oval.

	1	2	3	4	5	6	7	
Low	◯	◯	◯	◯	◯	◯	◯	Highest priority

63. Practice in a person-centered, humanistic way (empathy, compassion, person-centeredness) in the context of extensive use of AI and other technologies.

Mark only one oval.

	1	2	3	4	5	6	7	
Low	◯	◯	◯	◯	◯	◯	◯	Highest priority

Data Science

Using a scale of 1 to 7, where 1 indicates lowest priority and 7 represents highest priority, assign a priority for each objective listed below.

64. Describe the role of large data and data science in health care, and the use of AI technologies to support their applications.

Mark only one oval.

	1	2	3	4	5	6	7	
Low	◯	◯	◯	◯	◯	◯	◯	Highest priority

65. Identify types of healthcare data

Mark only one oval.

	1	2	3	4	5	6	7	
Low	◯	◯	◯	◯	◯	◯	◯	Highest priority

66. Evaluate the quality of data derived from large data, especially in the context of quality improvement and research.

Mark only one oval.

	1	2	3	4	5	6	7	
Low	◯	◯	◯	◯	◯	◯	◯	Highest priority

67. Apply data preprocessing for AI model development

Mark only one oval.

	1	2	3	4	5	6	7	
Low	◯	◯	◯	◯	◯	◯	◯	Highest priority

Limitations

Using a scale of 1 to 7, where 1 indicates lowest priority and 7 represents highest priority, assign a priority for each objective listed below.

68. Recognise errors and biases within AI-derived information

Mark only one oval.

	1	2	3	4	5	6	7	
Low	◯	◯	◯	◯	◯	◯	◯	Highest priority

69. Explain the strengths and weaknesses of AI technologies used in medical education and health care.

Mark only one oval.

	1	2	3	4	5	6	7	
Low	◯	◯	◯	◯	◯	◯	◯	Highest priority

70. Predict and prepare for potential opportunities and threats that AI technologies may create

Mark only one oval.

	1	2	3	4	5	6	7	
Low	◯	◯	◯	◯	◯	◯	◯	Highest priority

71. Implement strategies to mitigate risks and errors in AI-driven decision-making

Mark only one oval.

	1	2	3	4	5	6	7	
Low	◯	◯	◯	◯	◯	◯	◯	Highest priority

72. Participate in quality improvement, including incident reporting and analysis, related to the use of AI in clinical care.

Mark only one oval.

	1	2	3	4	5	6	7	
Low	◯	◯	◯	◯	◯	◯	◯	Highest priority

73. Apply techniques and strategies to enhance the use of AI technologies and mitigate their limitations.

Mark only one oval.

	1	2	3	4	5	6	7	
Low	◯	◯	◯	◯	◯	◯	◯	Highest priority

Continuous Learning and Adaptation

Using a scale of 1 to 7, where 1 indicates lowest priority and 7 represents highest priority, assign a priority for each objective listed below.

74. Stay updated on the rapid evolution and advancement in AI technologies in health care, including clinical practice, learning and health services management.

Mark only one oval.

	1	2	3	4	5	6	7	
Low	◯	◯	◯	◯	◯	◯	◯	Highest priority

75. Maintain an ongoing commitment to learn about new AI tools, technologies and practices that emerge

Mark only one oval.

	1	2	3	4	5	6	7	
Low	◯	◯	◯	◯	◯	◯	◯	Highest priority

76. Adapt to new AI technologies and methodologies as they emerge

Mark only one oval.

	1	2	3	4	5	6	7	
Low	◯	◯	◯	◯	◯	◯	◯	Highest priority

77. Describe the use of AI, including generative AI, to support staying up to date with the literature.

Mark only one oval.

	1	2	3	4	5	6	7	
Low	◯	◯	◯	◯	◯	◯	◯	Highest priority

78. Use AI effectively to stay up to date with the literature.

Mark only one oval.

	1	2	3	4	5	6	7	
Low	◯	◯	◯	◯	◯	◯	◯	Highest priority

This content is neither created nor endorsed by Google.

Google Forms
