# Supplementary material for: What and when: a Delphi study identifying AI competencies and learning objectives for medical students
Source: Front Digit Health. 2026 Sep 30;8:1918847. doi: 10.3389/fdgth.2026.1918847 (PMC13662149; doi:10.3389/fdgth.2026.1918847)
Supplement: Supplementary file 2 [file Supplementaryfile4.pdf]

Round 3 - AI Learning Competencies

Survey goal:
To rate the importance of the following learning objectives for medical students to achieve upon graduation. Please indicate how essential each learning objective is for you, considering the knowledge, attitudes and skills you will have obtained.

Please set aside 20-30 minutes to answer the questions.

Please note that the objectives have been arranged in temporary categories. We will later collectively confirm the final categories as well as competencies.

* Indicates required question

1. Email *

Foundational information

Using a scale of 1 to 7, where 1 indicates lowest priority and 7 represents highest priority, assign a priority for each objective listed below.

2. Describe an overview of how AI systems are "trained" (e.g., supervised, unsupervised, and reinforcement learning). *

Mark only one oval.

	1	2	3	4	5	6	7	
lowe	◯	◯	◯	◯	◯	◯	◯	highest priority

3. Define what AI is. *

Mark only one oval.

	1	2	3	4	5	6	7	
lowe	◯	◯	◯	◯	◯	◯	◯	highest priority

4. Define and differentiate between concepts related to AI, such as traditional AI, machine learning (ML), deep learning (DL), large language modeling (LLM), and generative AI. *

Mark only one oval.

	1	2	3	4	5	6	7	
lowe	◯	◯	◯	◯	◯	◯	◯	highest priority

5. Name the different types of AI technologies, specifically as they relate to medicine (e.g. large language model agents) *

Mark only one oval.

	1	2	3	4	5	6	7	
lowe	◯	◯	◯	◯	◯	◯	◯	highest priority

6. Select the most appropriate AI technology for the specific task or need. *

Mark only one oval.

	1	2	3	4	5	6	7	
lowe	◯	◯	◯	◯	◯	◯	◯	highest priority

7. Provide examples of the applications of AI in the various fields of medicine (e.g. clinical diagnostics and decision-making, health records management, screening, education, research). *

Mark only one oval.

	1	2	3	4	5	6	7	
lowe	◯	◯	◯	◯	◯	◯	◯	highest priority

8. Create effective prompts for ChatGPT or other AI-generative platforms. *

Mark only one oval.

	1	2	3	4	5	6	7	
lowe	◯	◯	◯	◯	◯	◯	◯	highest priority

9. Would you like to add or change anything, or do you have any comments about this section?

AI in Learning

Using a scale of 1 to 7, where 1 indicates lowest priority and 7 represents highest priority, assign a priority for each objective listed below.

10. Describe how AI can support and undermine learning (as a medical student) in general. *

Mark only one oval.

	1	2	3	4	5	6	7	
lowe	◯	◯	◯	◯	◯	◯	◯	highest priority

11. Use AI effectively, professionally, and ethically to support medical learning in UGME. (undergraduate medical education) *

Mark only one oval.

	1	2	3	4	5	6	7	
lowe	◯	◯	◯	◯	◯	◯	◯	highest priority

12. Identify the limits of AI in support of learning (when should we use it and when should we not use it.) *

Mark only one oval.

	1	2	3	4	5	6	7	
lowe	◯	◯	◯	◯	◯	◯	◯	highest priority

13. Would you like to add or change anything, or do you have any comments about this section?

Clinical applications of AI

Using a scale of 1 to 7, where 1 indicates lowest priority and 7 represents highest priority, assign a priority for each objective listed below.

14. Describe clinical applications of AI. *

Mark only one oval.

1	2	3	4	5	6	7
◯	◯	◯	◯	◯	◯	◯

15. Describe ethical risks or pitfalls related to using AI in clinical practice. *

Mark only one oval.

1	2	3	4	5	6	7
◯	◯	◯	◯	◯	◯	◯

16. Describe strategies to avoid risks and pitfalls related to using AI in clinical practice. *

Mark only one oval.

1	2	3	4	5	6	7
◯	◯	◯	◯	◯	◯	◯

17. Collaborate effectively with AI systems in clinical workflows. *

Mark only one oval.

1	2	3	4	5	6	7
◯	◯	◯	◯	◯	◯	◯

18. Describe the use of AI technologies to support clinical workflows (such as documenting patient visits in electronic health records, optimizing clinical workflow, etc.) *

Mark only one oval.

1	2	3	4	5	6	7
◯	◯	◯	◯	◯	◯	◯

19. Describe the application of AI technologies in clinical settings, including their roles in diagnostics, treatment planning, patient monitoring, and risk assessment. *

Mark only one oval.

1	2	3	4	5	6	7
◯	◯	◯	◯	◯	◯	◯

20. Describe how AI could enhance or undermine clinical reasoning. *

Mark only one oval.

1	2	3	4	5	6	7
◯	◯	◯	◯	◯	◯	◯

21. Use information and data derived from AI in combination with other reliable sources of medical information to aid in clinical care and learning. *

Mark only one oval.

1	2	3	4	5	6	7
◯	◯	◯	◯	◯	◯	◯

22. Learn how to apply critical thinking when using and applying AI output in practice. *

Mark only one oval.

1	2	3	4	5	6	7
◯	◯	◯	◯	◯	◯	◯

23. Analyze information and data obtained by AI in healthcare. *

Mark only one oval.

1	2	3	4	5	6	7
◯	◯	◯	◯	◯	◯	◯

24. Inform patients and care providers about the use of AI in health care, including its strengths and limitations, when such technology is used to support their care (e.g., helping make care decisions). *

Mark only one oval.

1	2	3	4	5	6	7
◯	◯	◯	◯	◯	◯	◯

25. Respond to patients in a way that demonstrates collaborative, shared decision-making and best evidence-driven care when they approach you with information they found using AI. *

Mark only one oval.

1	2	3	4	5	6	7
◯	◯	◯	◯	◯	◯	◯

26. Differentiate AI from augmented reality (AR) and virtual reality (VR) regarding their use in health care. *

Mark only one oval.

	1	2	3	4	5	6	7	
lowe	◯	◯	◯	◯	◯	◯	◯	highest priority

27. Would you like to add or change anything, or do you have any comments about this section?

AI application in medical research

Using a scale of 1 to 7, where 1 indicates lowest priority, and 7 represents highest priority, assign a priority for each objective listed below.

28. Describe the effective and ethical use of AI in medical research. *

Mark only one oval.

1	2	3	4	5	6	7
◯	◯	◯	◯	◯	◯	◯

29. Describe the ethical challenges and limitations of AI in medical research and strategies to mitigate these. *

Mark only one oval.

1	2	3	4	5	6	7
◯	◯	◯	◯	◯	◯	◯

30. Identify the opportunities and threats of the use of AI in medical research *

Mark only one oval.

1	2	3	4	5	6	7
◯	◯	◯	◯	◯	◯	◯

31. Use AI to enhance the critical appraisal of literature (studies) *

Mark only one oval.

1	2	3	4	5	6	7
◯	◯	◯	◯	◯	◯	◯

32. Would you like to add or change anything, or do you have any comments about this section?

____
____
____
____
____

AI applications in health records, data and practice management

Using a scale of 1 to 7, where 1 indicates lowest priority and 7 represents highest priority, assign a priority for each objective listed below.

33. Describe AI technology in support of medical record keeping and retrieval of medical records. *

Mark only one oval.

1	2	3	4	5	6	7
◯	◯	◯	◯	◯	◯	◯

34. Describe AI technology to support quality improvement using electronic health records. *

Mark only one oval.

1	2	3	4	5	6	7
◯	◯	◯	◯	◯	◯	◯

35. Describe the quality of data derived from large data, especially in the context of quality improvement and research. *

Mark only one oval.

1	2	3	4	5	6	7
◯	◯	◯	◯	◯	◯	◯

36. Would you like to add or change anything, or do you have any comments about this section?

____
____
____
____
____

Teamwork and interdisciplinary collaboration

Using a scale of 1 to 7, where 1 indicates lowest priority, and 7 represents highest priority, assign a priority for each objective listed below.

37. Apply a multidisciplinary approach when adopting or using AI in clinical practice, including collaborating with data scientists, computer engineers, biomedical engineers, and other experts. *

Mark only one oval.

1	2	3	4	5	6	7
◯	◯	◯	◯	◯	◯	◯

38. Participate in and contribute medical expertise to interdisciplinary AI projects on health care delivery and health-related education. *

Mark only one oval.

1	2	3	4	5	6	7
◯	◯	◯	◯	◯	◯	◯

39. Would you like to add or change anything, or do you have any comments about this section?

Legal implications and considerations

Using a scale of 1 to 7, where 1 indicates lowest priority, and 7 represents highest priority, assign a priority for each objective listed below.

40. Explain the legal implications of AI usage, including potential risks. *

Mark only one oval.

1	2	3	4	5	6	7
◯	◯	◯	◯	◯	◯	◯

41. Describe best practices and regulatory requirements for data protection, privacy, anonymity, and consent associated with using AI in healthcare. *

Mark only one oval.

1	2	3	4	5	6	7
◯	◯	◯	◯	◯	◯	◯

42. Would you like to add or change anything, or do you have any comments about this section?

Ethical and Professional Use of AI

Using a scale of 1 to 7, where 1 indicates lowest priority, and 7 represents highest priority, assign a priority for each objective listed below.

43. Identify ethical considerations when using AI technologies for learning, medical practice, health records keeping and analyses, research, and quality improvement. *

Mark only one oval.

1	2	3	4	5	6	7
◯	◯	◯	◯	◯	◯	◯

44. Use AI technologies ethically and professionally to support learning in medical education, clinical care, administration, research, and quality improvement. *

Mark only one oval.

1	2	3	4	5	6	7
◯	◯	◯	◯	◯	◯	◯

45. Describe under which circumstances healthcare professionals must practice transparency and professionalism by acknowledging the use of AI-generated information sources in medical education, research, and clinical practice. *

Mark only one oval.

1	2	3	4	5	6	7
◯	◯	◯	◯	◯	◯	◯

46. Apply AI technologies effectively when they are used to aid in clinical care, including analyzing results and making decisions. *

Mark only one oval.

1	2	3	4	5	6	7
◯	◯	◯	◯	◯	◯	◯

47. Practice in a person-centered, humanistic way (empathy, compassion, person-centeredness) in the context of extensive use of AI and other technologies. *

Mark only one oval.

1	2	3	4	5	6	7
◯	◯	◯	◯	◯	◯	◯

48. Would you like to add or change anything, or do you have any comments about this section?

____
____
____
____
____

Limitations/Strengths

Using a scale of 1 to 7, where 1 indicates lowest priority, and 7 represents highest priority, assign a priority for each objective listed below.

49. Recognize errors and biases within AI-derived information. *

Mark only one oval.

1	2	3	4	5	6	7
◯	◯	◯	◯	◯	◯	◯

50. Explain the strengths and weaknesses of AI technologies used in medical education. *

Mark only one oval.

1	2	3	4	5	6	7
◯	◯	◯	◯	◯	◯	◯

51. Explain the strengths and weaknesses of AI technologies used in health care. *

Mark only one oval.

1	2	3	4	5	6	7
◯	◯	◯	◯	◯	◯	◯

52. Predict and prepare for potential opportunities and threats that AI technologies may create. *

Mark only one oval.

1	2	3	4	5	6	7
◯	◯	◯	◯	◯	◯	◯

53. Implement strategies to mitigate risks and the use of AI technology in medical education. *

Mark only one oval.

1	2	3	4	5	6	7
◯	◯	◯	◯	◯	◯	◯

54. Would you like to add or change anything, or do you have any comments about this section?

Continuous Learning and Adaptation

Using a scale of 1 to 7, where 1 indicates lowest priority, and 7 represents highest priority, assign a priority for each objective listed below.

55. Stay updated on AI technologies' rapid evolution and advancement in health care, including clinical practice, learning, and health services management. *

Mark only one oval.

1	2	3	4	5	6	7
◯	◯	◯	◯	◯	◯	◯

56. Maintain an ongoing commitment to learning about emerging AI tools, technologies, and practices. *

Mark only one oval.

1	2	3	4	5	6	7
◯	◯	◯	◯	◯	◯	◯

57. Adapt to new AI technologies and methodologies as they emerge. *

Mark only one oval.

1	2	3	4	5	6	7
◯	◯	◯	◯	◯	◯	◯

58. Use AI effectively to stay up to date with the literature. *

Mark only one oval.

1	2	3	4	5	6	7
◯	◯	◯	◯	◯	◯	◯

59. Would you like to add or change anything, or do you have any comments about this section?

This content is neither created nor endorsed by Google.

Google Forms
