# Supplementary material for: What and when: a Delphi study identifying AI competencies and learning objectives for medical students
Source: Front Digit Health. 2026 Sep 30;8:1918847. doi: 10.3389/fdgth.2026.1918847 (PMC13662149; doi:10.3389/fdgth.2026.1918847)
Supplement: Supplementary file 3 [file Supplementaryfile5.pdf]

Competencies and Learning Objectives for Undergraduate Medical Education

For this study, we distinguish between **"Competencies"** and **"Learning Objectives"** as follows:

- **Competencies**: are the primary or overall abilities students should acquire, nurture, and demonstrate. We think of them as overall curriculum goals. Each Competency, therefore, has several enabling or supporting Learning Objectives
- **Learning Objectives**: These are the learner-centered, specific knowledge, skills, and attitudes that collectively support or enable the Competencies. Multiple enabling Learning Objectives support each Competency.

While we will ask you to comment on the overall Competencies we have identified, we **wish to focus mainly on the Learning Objectives**. We tried to identify learning objectives (and then the competencies that arise from these) that are appropriate, feasible and doable at a level that is expected upon completing undergraduate medical education. We anticipate that following undergraduate education, the students will continue learning and applying AI more during their postgraduate training (residency training). We do not cover postgraduate training in this work.

With that background, this survey is divided into two parts:

Part 1: A list of the overall Competencies that we have identified, informed by the Learning Objectives we developed.

Part 2: The list of Learning Objectives, organized by categories (not Competencies), we have identified.

* Indicates required question

1. Email *

____

Part 1. You will find below a list of overarching competencies. If you agree with them all, please select the option "Agree". If you feel that changes are needed in one or more of the overarching competencies (e.g. discard, change wording, change level to be more appropriate for undergraduate medical training) or if you wish to add a new one(s), please write them in the space provided.

2. **COMPETENCIES** *

1. Define and describe artificial intelligence (AI)

2. Use AI effectively and professionally to support learning in medicine.

3. Explain the role of AI in supporting clinical care, research and quality improvement.

4. Apply best ethical and legal practices when using AI to support learning and clinical care.

5. Collaborate with other disciplines when participating in AI-related initiatives and projects.

6. Update oneself continuously on emerging AI technologies, trends and applications.

Mark only one oval.

- ◯ AGREE
- ◯ Generally AGREE, but suggest some changes as follows
- ◯ DISAGREE (Please explain reasons for this below)

3. **Please explain if you selected "Agree, with some changes" or "Disagree."**

____
____
____
____
____

4. **Please feel free to add any additional thoughts (e.g. overall thoughts on this work, additional Competencies), ideas, or links to resources you think would be useful.**

Part 2. LEARNING OBJECTIVES

The learning objectives are organized by category in the following sections. There are ten categories with a total of 35 learning objectives. For each learning objective, indicate whether you agree, generally agree, or disagree.

There is a text box after each learning competency for you to provide additional thoughts. If you generally agree but with suggestions or disagree a learning objective, please explain why in the space provided below each learning objective.

At the end of each section, there is an additional optional textbox that allows you to add additional learning objectives not listed that you think are important.

1. Foundational information

5. **Define what AI is.** *

Mark only one oval.

- AGREE
- Generally AGREE, but suggest some changes as follows
- DISAGREE (Please explain reasons for this below)

6. **Please explain if you selected "Agree, with some changes" or "Disagree."**

7. **Describe how AI systems are "trained" (e.g., supervised, unsupervised, and reinforcement learning).** *

Mark only one oval.

- AGREE
- Generally AGREE, but suggest some changes as follows
- DISAGREE (Please explain reasons for this below)

8. **Please explain if you selected "Agree, with some changes" or "Disagree."**

9. **Define and differentiate between concepts related to AI, such as traditional AI, machine learning (ML), deep learning (DL), large language modeling (LLM), generative AI, augmented reality, and virtual reality.** *

Mark only one oval.

- AGREE
- Generally AGREE, but suggest some changes as follows
- DISAGREE (Please explain reasons for this below)

10. **Please explain if you selected "Agree, with some changes" or "Disagree."**

11. **Select the most appropriate AI technology for the specific task or need.** *

Mark only one oval.

- AGREE
- Generally AGREE, but suggest some changes as follows
- DISAGREE (Please explain reasons for this below)

12. **Please explain if you selected "Agree, with some changes" or "Disagree."**

13. **Provide examples of the applications of AI in the various fields of medicine (e.g., clinical diagnostics and decision-making, health records management, screening, education, and research).** *

Mark only one oval.

- AGREE
- Generally AGREE, but suggest some changes as follows
- DISAGREE (Please explain reasons for this below)

14. **Please explain if you selected "Agree, with some changes" or "Disagree."**

15. **Create effective prompts for ChatGPT or other AI-generative platforms.** *

Mark only one oval.

- AGREE
- Generally AGREE, but suggest some changes as follows
- DISAGREE (Please explain reasons for this below)

16. **Please explain if you selected "Agree, with some changes" or "Disagree."**

17. **Recognize errors and biases within AI-derived information.** *

Mark only one oval.

- AGREE
- Generally AGREE, but suggest some changes as follows
- DISAGREE (Please explain reasons for this below)

18. **Please explain if you selected "Agree, with some changes" or "Disagree."**

19. **Please feel free to add any additional thoughts (e.g. overall thoughts on this work, additional learning objectives), ideas, or links to resources you think would be useful.**

2. AI in Learning

20. **Describe how AI can support and undermine learning (as a medical student).** *

Mark only one oval.

- AGREE
- Generally AGREE, but suggest some changes as follows
- DISAGREE (Please explain reasons for this below)

21. **Please explain if you selected "Agree, with some changes" or "Disagree."**

22. **Use AI effectively, professionally, and ethically to support medical learning in UGME. (Undergraduate Medical Education)** *

Mark only one oval.

- AGREE
- Generally AGREE, but suggest some changes as follows
- DISAGREE (Please explain reasons for this below)

23. **Please explain if you selected "Agree, with some changes" or "Disagree."**

24. **Describe how AI could enhance or undermine clinical reasoning.** *

Mark only one oval.

- AGREE
- Generally AGREE, but suggest some changes as follows
- DISAGREE (Please explain reasons for this below)

25. **Please explain if you selected "Agree, with some changes" or "Disagree."**

26. **Apply critical thinking when analyzing AI-generated information in medical learning.** *

Mark only one oval.

- AGREE
- Generally AGREE, but suggest some changes as follows
- DISAGREE (Please explain reasons for this below)

27. **Please explain if you selected "Agree, with some changes" or "Disagree."**

28. **Please feel free to add any additional thoughts (e.g. overall thoughts on this work, additional learning objectives), ideas, or links to resources you think would be useful.**

3. Clinical applications of AI

29. **Describe ethical risks or pitfalls related to using AI in clinical practice.** *

Mark only one oval.

- AGREE
- Generally AGREE, but suggest some changes as follows
- DISAGREE (Please explain reasons for this below)

30. **Please explain if you selected "Agree, with some changes" or "Disagree."**

31. **Describe strategies to avoid risks and pitfalls related to using AI in clinical practice.** *

Mark only one oval.

- AGREE
- Generally AGREE, but suggest some changes as follows
- DISAGREE (Please explain reasons for this below)

32. **Please explain if you selected "Agree, with some changes" or "Disagree."**

33. **Describe the use of AI to support clinical workflows, documentation, and scheduling.** *

Mark only one oval.

- AGREE
- Generally AGREE, but suggest some changes as follows
- DISAGREE (Please explain reasons for this below)

34. **Please explain if you selected "Agree, with some changes" or "Disagree."**

35. **Describe the application of AI technologies in clinical settings, including their roles in diagnostics, treatment planning, patient monitoring, and risk assessment.** *

Mark only one oval.

- AGREE
- Generally AGREE, but suggest some changes as follows
- DISAGREE (Please explain reasons for this below)

36. **Please explain if you selected "Agree, with some changes" or "Disagree."**

37. **Apply critical thinking when analyzing AI-generated information in clinical practice.** *

Mark only one oval.

- AGREE
- Generally AGREE, but suggest some changes as follows
- DISAGREE (Please explain reasons for this below)

38. **Please explain if you selected "Agree, with some changes" or "Disagree."**

39. **Respond to patients in a way that demonstrates collaborative, shared decision-making and best evidence-driven care when they approach you with information they found using AI.** *

Mark only one oval.

- AGREE
- Generally AGREE, but suggest some changes as follows
- DISAGREE (Please explain reasons for this below)

40. **Please explain if you selected "Agree, with some changes" or "Disagree."**

41. **Please feel free to add any additional thoughts (e.g. overall thoughts on this work, additional learning objectives), ideas, or links to resources you think would be useful.**

4. AI application in medical research

42. **Provide examples of how AI can assist in medical research.** *

Mark only one oval.

- AGREE
- Generally AGREE, but suggest some changes as follows
- DISAGREE (Please explain reasons for this below)

43. **Please explain if you selected "Agree, with some changes" or "Disagree."**

44. **Use AI effectively and appropriately to assist with literature searches and critically appraise articles.** *

Mark only one oval.

- AGREE
- Generally AGREE, but suggest some changes as follows
- DISAGREE (Please explain reasons for this below)

45. **Please explain if you selected "Agree, with some changes" or "Disagree."**

46. **Please feel free to add any additional thoughts (e.g. overall thoughts on this work, additional learning objectives), ideas, or links to resources you think would be useful.**

5. AI applications in health records, data, and practice management

47. **Provide examples of how AI can support quality improvement, including using data in health records to identify opportunities for improvement.** *

Mark only one oval.

- AGREE
- Generally AGREE, but suggest some changes as follows
- DISAGREE (Please explain reasons for this below)

48. **Please explain if you selected "Agree, with some changes" or "Disagree."**

49. **Describe the role of large data in supporting quality improvement and research.** *

Mark only one oval.

- AGREE
- Generally AGREE, but suggest some changes as follows
- DISAGREE (Please explain reasons for this below)

50. **Please explain if you selected "Agree, with some changes" or "Disagree."**

51. **Please feel free to add any additional thoughts (e.g. overall thoughts on this work, additional learning objectives), ideas, or links to resources you think would be useful.**

6. Teamwork and interdisciplinary collaboration

52. **Apply a multidisciplinary approach when adopting or using AI in clinical practice, including collaborating with data scientists, computer engineers, biomedical engineers, and other experts.** *

Mark only one oval.

- AGREE
- Generally AGREE, but suggest some changes as follows
- DISAGREE (Please explain reasons for this below)

53. **Please explain if you selected "Agree, with some changes" or "Disagree."**

54. **Describe the importance of a multidisciplinary approach, including the role of physicians, when undertaking AI initiatives in health care delivery and health-related education.** *

Mark only one oval.

- AGREE
- Generally AGREE, but suggest some changes as follows
- DISAGREE (Please explain reasons for this below)

55. **Please explain if you selected "Agree, with some changes" or "Disagree."**

56. **Please feel free to add any additional thoughts (e.g. overall thoughts on this work, additional learning objectives), ideas, or links to resources you think would be useful.**

7.Legal implications and considerations

57. **Explain the legal implications of AI use in health care, including potential risks and methods to mitigate these.** *

Mark only one oval.

- AGREE
- Generally AGREE, but suggest some changes as follows
- DISAGREE (Please explain reasons for this below)

58. **Please explain if you selected "Agree, with some changes" or "Disagree."**

59. **Describe best practices and regulatory requirements for data protection, privacy, anonymity, and consent for using AI in healthcare.** *

Mark only one oval.

- AGREE
- Generally AGREE, but suggest some changes as follows
- DISAGREE (Please explain reasons for this below)

60. **Please explain if you selected "Agree, with some changes" or "Disagree."**

61. **Please feel free to add any additional thoughts (e.g. overall thoughts on this work, additional learning objectives), ideas, or links to resources you think would be useful.**

8. Ethical and Professional Use of AI

62. **Describe the ethical considerations when using AI for learning, medical practice, health records keeping and analyses, research, and quality improvement.** *

Mark only one oval.

- AGREE
- Generally AGREE, but suggest some changes as follows
- DISAGREE (Please explain reasons for this below)

63. **Please explain if you selected "Agree, with some changes" or "Disagree."**

64. **Practice professionalism by acknowledging the use of AI when using technology in medical education, scholarship, and submitting assignments.** *

Mark only one oval.

- AGREE
- Generally AGREE, but suggest some changes as follows
- DISAGREE (Please explain reasons for this below)

65. **Please explain if you selected "Agree, with some changes" or "Disagree."**

66. **Practice in a person-centered, humanistic way (empathy, compassion, person-centeredness) in the context of extensive use of AI and other technologies.** *

Mark only one oval.

- AGREE
- Generally AGREE, but suggest some changes as follows
- DISAGREE (Please explain reasons for this below)

67. **Please explain if you selected "Agree, with some changes" or "Disagree."**

68. **Be transparent with patients and care providers when AI supports their clinical care and management (e.g., making diagnostic or treatment decisions)** *

Mark only one oval.

- AGREE
- Generally AGREE, but suggest some changes as follows
- DISAGREE (Please explain reasons for this below)

69. **Please explain if you selected "Agree, with some changes" or "Disagree."**

70. **Please feel free to add any additional thoughts (e.g. overall thoughts on this work, additional learning objectives), ideas, or links to resources you think would be useful.**

9. Limitations/Strengths

71. **Explain the strengths and weaknesses of AI technologies used in medical education.** *

Mark only one oval.

- AGREE
- Generally AGREE, but suggest some changes as follows
- DISAGREE (Please explain reasons for this below)

72. **Please explain if you selected "Agree, with some changes" or "Disagree."**

73. **Explain the strengths and weaknesses of AI technologies used in health care.** *

Mark only one oval.

- AGREE
- Generally AGREE, but suggest some changes as follows
- DISAGREE (Please explain reasons for this below)

74. **Please explain if you selected "Agree, with some changes" or "Disagree."**

75. **Predict and prepare for potential opportunities and threats that AI technologies may create.** *

Mark only one oval.

- AGREE
- Generally AGREE, but suggest some changes as follows
- DISAGREE (Please explain reasons for this below)

76. **Please explain if you selected "Agree, with some changes" or "Disagree."**

77. **Implement strategies to mitigate risks and the use of AI technology in medical education.** *

Mark only one oval.

- AGREE
- Generally AGREE, but suggest some changes as follows
- DISAGREE (Please explain reasons for this below)

78. **Please explain if you selected "Agree, with some changes" or "Disagree."**

79. **Please feel free to add any additional thoughts (e.g. overall thoughts on this work, additional learning objectives), ideas, or links to resources you think would be useful.**

10. Continuous Learning and Adaptation

80. **Commit to ongoing learning and updating on emerging AI technologies and practices in health care.** *

Mark only one oval.

- AGREE
- Generally AGREE, but suggest some changes as follows
- DISAGREE (Please explain reasons for this below)

81. **Please explain if you selected "Agree, with some changes" or "Disagree."**

82. **Adapt to new AI technologies and methodologies as they emerge.** *

Mark only one oval.

- AGREE
- Generally AGREE, but suggest some changes as follows
- DISAGREE (Please explain reasons for this below)

83. **Please explain if you selected "Agree, with some changes" or "Disagree."**

84. **Please feel free to add any additional thoughts (e.g. overall thoughts on this work, additional learning objectives), ideas, or links to resources you think would be useful.**

This content is neither created nor endorsed by Google.

Google Forms
