# Supplementary material for: What and when: a Delphi study identifying AI competencies and learning objectives for medical students
Source: Front Digit Health. 2026 Sep 30;8:1918847. doi: 10.3389/fdgth.2026.1918847 (PMC13662149; doi:10.3389/fdgth.2026.1918847)
Supplement: Supplementary file 4 [file Supplementaryfile1.docx]

# **Supplement 1. DELPHISTAR reporting checklist**

“Reporting items follow the DELPHISTAR guideline: Niederberger M, Schifano J, Deckert S, et al. Delphi studies in social and health sciences—Recommendations for an interdisciplinary standardized reporting (DELPHISTAR). PLoS One. 2024;19(8):e0304651. doi:10.1371/journal.pone.0304651

| **Item** | **Section** | **Checklist item** | **Location in manuscript / notes** |
| --- | --- | --- | --- |
| 1 | Title | Identification as a Delphi study in the title | Title |
| 2 | Abstract | Identification as a Delphi study in the abstract | Abstract, Methods (“A four-round Delphi process was conducted…”). |
| 3 | Abstract | Structured abstract | Abstract is structured (Background, Methods, Results, Conclusion). |
| 4 | Formal | Information about the sources of funding | Declarations, Funding (“no support from any organization”). |
| 5 | Formal | Information about the team of authors/researchers (e.g., discipline, institution) | Title-page affiliations; Methods (Medical Education Unit; students and educators). |
| 6 | Formal | Information about method consulting | Methods |
| 7 | Formal | Information about the project background | Methods (broader co-design initiative: scoping review, qualitative study, two Delphi studies). |
| 8 | Formal | Information about the study protocol | Available upon request |
| 9 | Content | Justification of the chosen method (Delphi) to answer the research question | Background / Methods (rationale for a modified, co-design Delphi to build stakeholder consensus). |
| 10 | Content | Aim of the Delphi study (e.g., consensus, forecasting) | Background; Methods (aim = consensus on AI competencies and LOs and their timing). |
| 11 | Body & integration of knowledge | Identification and elucidation of relevant expertise, spheres of experience and perspectives | Methods, Participants; Supplement 2 (expert domains and criteria). |
| 12 | Body & integration of knowledge | Handling of knowledge  /expertise/perspectives that are missing or deliberately not integrated | Limitations |
| 13 | Body & integration of knowledge | Basic definition of expert | Supplement 2 (definition and eligibility criteria); Methods, Participants. |
| 14 | Delphi variant & modifications | Identification of the type of Delphi variant and potential modifications | Methods (“modified, four-round Delphi” incorporating students and a separate expert round). |
| 15 | Delphi variant & modifications | Justification of the Delphi variant and modifications, including during the study | Methods (rationale for student inclusion and for adding the external-expert validation round). |
| 16 | Sample of experts | Selection criteria for the experts (per round, per group if applicable) | Methods, Participants; Supplement 2 (Round 4 panel criteria). |
| 17 | Sample of experts | Identification of the experts | Supplement 2 (panel composition, roles and countries). |
| 18 | Sample of experts | Information about recruiting and any subsequent recruiting of experts | Methods (first-year cohort invited to the core panel; experts invited for Round 4). |
| 19 | Survey | Elucidation of the content development for the questionnaire | Methods, Round 1 (in-person generation; informed by the scoping review and orientation). |
| 20 | Survey | Description of the questionnaire (content and structure) | Methods, Rounds 2–4; Supplements 3–5 (full questionnaires). |
| 21 | Delphi rounds | Number of Delphi rounds | Methods (four rounds); Figure 1. |
| 22 | Delphi rounds | Information about the aims of the individual Delphi rounds | Methods, Round 1–4 (objective stated for each round). |
| 23 | Delphi rounds | Disclosure and justification of the criterion for discontinuation | The four-round design was planned |
| 24 | Feedback | Information about what data was reported back per round | Methods, Rounds 2–3 (anonymized statistical summaries shared in an online forum). |
| 25 | Feedback | How the previous round’s results were fed back (frequencies, means, dispersion, comments) | Methods, Data analysis (median, IQR and participants’ comments fed back). |
| 26 | Feedback | Whether feedback was differentiated by specific groups | A single panel was used and feedback was aggregated, not group-differentiated. |
| 27 | Feedback | How dissent and unclear results were handled | Methods, Data analysis (structured qualitative review of dispersed items → reword/merge/exclude). |
| 28 | Data analysis | Disclosure of the quantitative and qualitative analytical strategy | Methods, Data analysis and consensus criteria (descriptive statistics + thematic review of comments). |
| 29 | Data analysis | Definition and measurement of consensus | Methods, Data analysis (retention rule: median ≥ 6 and IQR ≤ 2). |
| 30 | Data analysis | Group-specific analysis or weighting of experts | No expert weighting or group-specific analysis was applied. |
| 31 | Delphi process | Illustration of the Delphi study (e.g., flow chart) | Figure 1 (Delphi overview); item-flow Supplement 6 (Delphi evolution). |
| 32 | Delphi process | Information about special aspects/deviations during the study (with justification) | Methods – Study design and overview |
| 33 | Delphi process | Number of experts per round (both invited and participating) | Results/Methods (Round 2: 15/16, 94%; Round 3: 16/16, 100%; Round 4: 10/10). |
| 34 | Results | Presentation of the results for each Delphi round and the final results | Results (per-round narrative); Table 1; DelphiEvolution Supplement 6 (per-round dispositions). |
| 35 | Quality of findings | Highlighting the findings from the Delphi study | Discussion; Conclusions. |
| 36 | Quality of findings | Validity of the results (e.g., transferability of the findings) | Discussion; Limitations (international panel; may not transfer to all contexts). |
| 37 | Quality of findings | Reliability of the results (e.g., split half, inter-rater reliability) | Not applicable |
| 38 | Quality of findings | Reflection on potential limitations (e.g., number of experts, response bias) | Limitations (relatively small group of 26; single-institution core panel; generalizability). |
