# Supplementary material for: What and when: a Delphi study identifying AI competencies and learning objectives for medical students
Source: Front Digit Health. 2026 Sep 30;8:1918847. doi: 10.3389/fdgth.2026.1918847 (PMC13662149; doi:10.3389/fdgth.2026.1918847)
Supplement: Supplementary file 5 [file Supplementaryfile2.docx]

# Supplement 2. Expert Panel and Eligibility Criteria

This supplement specifies the criteria for constituting the expert panel that validated the competencies and learning objectives in Round 4 of the Delphi process.

## Definition of an expert

This study concerns competencies and learning objectives for artificial intelligence (AI) in undergraduate medical education. Expertise was defined to capture two complementary domains: (i) substantive expertise in AI applied to medicine and medical education, and (ii) expertise in medical-education curriculum design and governance. A panelist was eligible if they met at least one of the four criteria below, and the panel was required to include both expertise domains listed above.

## Eligibility criteria

1. **Educational leadership and curriculum governance.** Holds, or has held, a formal academic leadership position with responsibility for the design, oversight, or governance of an undergraduate medical or health-professions curriculum (for example, Dean or Vice-Dean of a medical or dental school, Head or Coordinator of a faculty education office, or elected leadership of a national society for medical education).
2. **Scholarship or formal training in health-professions education.** Demonstrated expertise in health-professions education evidenced by a formal postgraduate qualification (e.g., a medical-education fellowship or diploma in clinical education), peer-reviewed educational scholarship, or an editorial role in a relevant medical-education or health-technology journal.
3. **Clinical practice with a teaching responsibility.** A licensed physician or dentist actively engaged in clinical practice and in the teaching, supervision, or training of undergraduate health-professions students.
4. **Demonstrated expertise in AI applied to medicine or health-professions education.** Evidenced by peer-reviewed publications or scholarship on AI in medicine or medical education, leadership of an AI-focused research program, or professional or enterprise engagement in developing or implementing AI tools for healthcare or medical education.

## Rationale for a mixed-expertise panel

The panel was deliberately heterogeneous. Validating AI competencies for an undergraduate medical curriculum requires both substantive AI knowledge and the educational judgment to determine what is appropriate for undergraduate learners; a single expertise profile would be insufficient for either task alone. This mixed-expertise design is consistent with established Delphi methodology for AI-in-medical-education competency studies. Çalışkan, Demir, and Karaca (2022) similarly constituted their e-Delphi panel from distinct expert backgrounds (healthcare professionals and academicians, computer and data science professionals, law and ethics professionals, and medical students) to capture the range of perspectives needed to define AI competencies for medical graduates

Table S2.1. Panel composition and criteria met

| **#** | **Panellist** | **Primary role / Country** | **C1** | **C2** | **C3** | **C4** |
| --- | --- | --- | --- | --- | --- | --- |
| 1 | Expert 1 | Physician, Assistant Clinical Professor | — | ✓ | ✓ | ✓ |
| 2 | Expert 2 | Physician, Clinical Assistant Professor | — | ✓ | ✓ | ✓ |
| 3 | Expert 3 | Physician, Internal Medicine | — | — | ✓ | ✓ |
| 4 | Expert 4 | Physician, Dean | ✓ | ✓ | ✓ | — |
| 5 | Expert 5 | Physician, Vice-Dean | ✓ | ✓ | ✓ | — |
| 6 | Expert 6 | Physician, Professor | — | — | ✓ | ✓ |
| 7 | Expert 7 | Physician, Dean | ✓ | — | ✓ | ✓ |
| 8 | Expert 8 | Professor | ✓ | ✓ | — | ✓ |
| 9 | Expert 9 | Dentist, Assistant Professor & Head of Education Office | ✓ | ✓ | ✓ | — |
| 10 | Expert 10 | Researcher in AI and medical education | — | ✓ | — | ✓ |

*C1 = educational leadership/curriculum governance; C2 = scholarship or formal training in health-professions education; C3 = clinical practice with a teaching responsibility; C4 = demonstrated expertise in AI applied to medicine or medical education. ✓ = criterion met; — = criterion not met. A panelist needs to meet at least two criteria to qualify.*
