# Supplementary material for: What and when: a Delphi study identifying AI competencies and learning objectives for medical students
Source: Front Digit Health. 2026 Sep 30;8:1918847. doi: 10.3389/fdgth.2026.1918847 (PMC13662149; doi:10.3389/fdgth.2026.1918847)
Supplement: Supplementary file 6 [file Supplementaryfile6.docx]

# **Supplement 6. - Evolution of candidate learning objectives across the Delphi process**

This supplement documents how candidate learning objectives (LOs) were generated, rated, merged, reworded, removed and validated across the four rounds of the modified Delphi study. It is intended to make the process transparent and reproducible, in line with recommendations to present results for each Delphi round and to illustrate item flow (DELPHISTAR items 31 and 34). Quantitative ratings used a 7-point Likert scale; the a priori consensus rule was a median ≥ 6 and an interquartile range (IQR) ≤ 2. Dispositions in Tables S2 and S3 are derived from the consensus rule together with the qualitative review of participants’ comments that guided rewording, merging and removal. Identifiers: items in Table S2 are cited as R2-1…R2-77, items in Table S3 as R3-1…R3-48, and final learning objectives as the Table 1 numbering of the manuscript (e.g., LO 2.2). These IDs make every objective traceable across rounds.

## **Table S1. Summary of item flow across the four Delphi rounds**

| **Round (date)** | **Activity** | **Items entering** | **Items exiting** | **Net change** | **Basis for change** |
| --- | --- | --- | --- | --- | --- |
| **Round 1 (May 2024)** | In-person idea generation (14 students + 2 educators); real-time compilation and thematic synthesis; secondary synthesis by lead investigators (June 2024) | LOs brought (8 per participant) plus items generated in session | 134 candidate LOs → 77 consolidated LOs | −57 | Real-time grouping of identical/similar statements; thematic analysis; rewriting of each representative LO for rating |
| **Round 2 (Sep 2024)** | Online rating, 7-point Likert (15/16 respondents; 94%) | 77 | 48 | −29 | A priori consensus rule (median ≥ 6 and IQR ≤ 2): 52 items had median ≥ 6 (46 met both criteria); 6 had high median but dispersed ratings (one IQR 3.5); 27 fell below median 6. After qualitative review of comments, merging of overlapping items, rewording, and removal of the lowest rated, 48 LOs advanced. |
| **Round 3 (Oct 2024)** | Online re-rating, 7-point Likert (16/16 respondents; 100%) | 48 | 35 | −13 | Same consensus rule applied unchanged: 42 items had median ≥ 6 (41 met both criteria); 1 had high median but dispersed ratings (IQR 3); 6 fell below median 6. After qualitative review, merging and rewording, items were reduced to 35 LOs and organized into 6 overarching competencies. |
| **Round 4 (Nov 2024–Jan 2025)** | External expert validation (n = 10; agree / agree-with-suggestions / disagree) followed by a final consensus meeting of the full team | 35 LOs / 6 competencies | 37 LOs / 7 competencies | +2 LOs / +1 competency | Unanimous agreement on appropriateness; 20 LOs accepted unchanged, 15 received suggestions; 4 new LOs proposed and 2 existing LOs merged (net +2); 2 competencies reworded and 1 new competency added. |

**Table S2. Round 2 (September 2024): ratings and disposition of the 77 candidate learning objectives**

*Ratings by the panel (15 of 16 responded; 94%) on a 7-point Likert scale. Items meeting the consensus rule were retained; high-median but dispersed items underwent qualitative review; items below median 6 were treated as low priority. The retained pool was subsequently reworded and merged to the 48 LOs that formed the Round 3 questionnaire.*

| **#** | **Learning objective (as rated)** | **Median** | **IQR** | **Disposition** | **Traceability: disposition after Round 2** |
| --- | --- | --- | --- | --- | --- |
| 1 | Practice in a person-centered, humanistic way (empathy, compassion, person-centeredness) in the context of extensive use of AI and other technologies. | 7.0 | 2.0 | Met consensus (median ≥ 6, IQR ≤ 2) — retained | Advanced → R3-11 (unchanged) |
| 2 | Create effective prompts for ChatGPT or other AI platforms | 7.0 | 1.5 | Met consensus (median ≥ 6, IQR ≤ 2) — retained | Advanced → R3-13 (reworded; R2-21 and R2-43 merged after qualitative review) |
| 3 | Describe ethical risks or pitfalls related to the use of AI in clinical practice. | 7.0 | 1.0 | Met consensus (median ≥ 6, IQR ≤ 2) — retained | Advanced → R3-23 (reworded) after qualitative review |
| 4 | Learn how to apply critical thinking when using and applying AI output in practice | 7.0 | 1.5 | Met consensus (median ≥ 6, IQR ≤ 2) — retained | Advanced → R3-4 (unchanged) |
| 5 | Identify the limits of AI in support of learning (when should we use it and when should we not use it | 7.0 | 0.5 | Met consensus (median ≥ 6, IQR ≤ 2) — retained | Advanced → R3-2 (unchanged) |
| 6 | Implement strategies to mitigate risks and errors in AI-driven decision-making | 7.0 | 1.5 | Met consensus (median ≥ 6, IQR ≤ 2) — retained | Advanced → R3-17 (reworded after qualitative review) |
| 7 | Use AI tools to enhance medical learning in a way that is effective and professional. | 7.0 | 1.0 | Met consensus (median ≥ 6, IQR ≤ 2) — retained | Merged into R3-1 (with R2-8) after qualitative review |
| 8 | Use AI effectively, professionally, and ethically in support of medical learning in UGME. (Undergraduate medical education) | 7.0 | 1.0 | Met consensus (median ≥ 6, IQR ≤ 2) — retained | Advanced → R3-1 (reworded) |
| 9 | Use information derived from AI in combination with other reliable sources of medical information to aid in clinical care and learning. | 7.0 | 2.0 | Met consensus (median ≥ 6, IQR ≤ 2) — retained | Advanced → R3-27 (reworded after qualitative review) |
| 10 | Apply a multidisciplinary approach when adopting or using AI in clinical practice, including collaborating with data scientists, computer engineers, biomedical engineers, and other experts | 7.0 | 3.5 | High median but dispersed (IQR > 2) — qualitative review | Retained after qualitative review → R3-6 (unchanged) |
| 11 | Provide examples of the applications of AI in the various fields of medicine (e.g. clinical diagnostics and decision-making, health records management, screening, education, research). | 7.0 | 2.0 | Met consensus (median ≥ 6, IQR ≤ 2) — retained | Advanced → R3-21 (unchanged) |
| 12 | Understand the ethical limits and considerations when employing AI | 7.0 | 1.5 | Met consensus (median ≥ 6, IQR ≤ 2) — retained | Merged into R3-36 after qualitative review |
| 13 | Discuss the ethical implications of using AI (consequences) | 7.0 | 1.0 | Met consensus (median ≥ 6, IQR ≤ 2) — retained | Merged into R3-36 after qualitative review |
| 14 | Describe the effective and ethical use of AI in medical research | 7.0 | 1.5 | Met consensus (median ≥ 6, IQR ≤ 2) — retained | Advanced → R3-30 (unchanged) |
| 15 | Practice ethically and professionally using AI technologies to support learning in medical education, clinical care, administration, research, and quality improvement. | 7.0 | 1.0 | Met consensus (median ≥ 6, IQR ≤ 2) — retained | Advanced → R3-8 (reworded) |
| 16 | Recognize errors and biases within AI-derived information | 7.0 | 1.5 | Met consensus (median ≥ 6, IQR ≤ 2) — retained | Advanced → R3-12 (unchanged) |
| 17 | Apply best ethical and professional practices (e.g. ethical frameworks) to AI-powered decision-making processes and tools. | 7.0 | 1.0 | Met consensus (median ≥ 6, IQR ≤ 2) — retained | Merged into R3-8 after qualitative review |
| 18 | Use AI technology in support of medical record keeping and retrieval of medical records. | 6.0 | 2.5 | High median but dispersed (IQR > 2) — qualitative review | Retained and reworded after qualitative review → R3-44 |
| 19 | Describe how AI systems are “trained” (e.g. supervised, unsupervised and reinforcement learning). | 6.0 | 1.5 | Met consensus (median ≥ 6, IQR ≤ 2) — retained | Advanced → R3-46 (reworded after qualitative review) |
| 20 | Analyze information and data obtained by AI in healthcare | 6.0 | 1.5 | Met consensus (median ≥ 6, IQR ≤ 2) — retained | Advanced → R3-28 (unchanged) |
| 21 | Create effective prompts for ChatGPT or other AI platforms .1 | 6.0 | 2.5 | High median but dispersed (IQR > 2) — qualitative review | Duplicate of R2-2; merged into R3-13 after qualitative review |
| 22 | Use AI technology to support quality improvement using electronic health records. | 6.0 | 2.0 | Met consensus (median ≥ 6, IQR ≤ 2) — retained | Advanced → R3-33 (reworded after qualitative review) |
| 23 | Collaborate with data scientists, engineers, and other AI experts in healthcare settings | 6.0 | 2.5 | High median but dispersed (IQR > 2) — qualitative review | Retained after qualitative review; merged into R3-6 |
| 24 | Participate in, and contribute medical expertise to, interdisciplinary AI projects related to the delivery of health care and health-related education. | 6.0 | 1.5 | Met consensus (median ≥ 6, IQR ≤ 2) — retained | Advanced → R3-35 (reworded after qualitative review) |
| 25 | Foster teamwork, including effective communication across disciplines, when designing and using AI | 6.0 | 2.0 | Met consensus (median ≥ 6, IQR ≤ 2) — retained | Merged into R3-35 After qualitative review |
| 26 | Predict and prepare for potential opportunities and threats that AI technologies may create | 6.0 | 2.0 | Met consensus (median ≥ 6, IQR ≤ 2) — retained | Advanced → R3-38 (unchanged) |
| 27 | Respond to patients in a way that demonstrates collaborative, shared decision-making and best evidence-driven care when they approach you with information they found using AI. | 6.0 | 1.0 | Met consensus (median ≥ 6, IQR ≤ 2) — retained | Advanced → R3-15 (reworded after qualitative review) |
| 28 | Describe data protection and privacy laws in a specific jurisdiction when using AI in clinical practice | 6.0 | 2.0 | Met consensus (median ≥ 6, IQR ≤ 2) — retained | Merged into R3-7 after qualitative review |
| 29 | Apply the use of AI technologies responsibly and effectively when they are being used to aid in clinical care, including analyzing results and decision-making | 6.0 | 2.0 | Met consensus (median ≥ 6, IQR ≤ 2) — retained | Advanced → R3-10 (reworded after qualitative review) |
| 30 | Explain the legal implications of AI usage, including potential risks. | 6.0 | 2.0 | Met consensus (median ≥ 6, IQR ≤ 2) — retained | Advanced → R3-16 (unchanged) |
| 31 | Identify ethical concerns when using AI technologies for learning, medical practice, health records keeping and analyses, research, and quality improvement. | 6.0 | 2.0 | Met consensus (median ≥ 6, IQR ≤ 2) — retained | Advanced → R3-36 (reworded after qualitative review) |
| 32 | Describe issues of privacy, anonymity, consent, bias, transparency, and accountability with respect to the use of AI in medical practice and research. | 6.0 | 1.5 | Met consensus (median ≥ 6, IQR ≤ 2) — retained | Merged into R3-7 after qualitative review |
| 33 | Explain the strengths and weaknesses of AI technologies used in medical education and health care. | 6.0 | 1.5 | Met consensus (median ≥ 6, IQR ≤ 2) — retained | Split → R3-45 (medical education) and R3-37 (health care) after qualitative review |
| 34 | Describe how AI could enhance or undermine clinical reasoning | 6.0 | 2.0 | Met consensus (median ≥ 6, IQR ≤ 2) — retained | Advanced → R3-3 (unchanged) |
| 35 | Identify the opportunities and threats of the use of AI in medical research | 6.0 | 1.5 | Met consensus (median ≥ 6, IQR ≤ 2) — retained | Advanced → R3-31 (unchanged) |
| 36 | Describe the strengths and limitations of AI systems in supporting clinical care, including in such as areas as critical thinking, and generating diagnoses and care plans. | 6.0 | 2.0 | Met consensus (median ≥ 6, IQR ≤ 2) — retained | Merged into R3-37 after qualitative review |
| 37 | Define what AI is. | 6.0 | 2.5 | High median but dispersed (IQR > 2) — qualitative review | Retained after qualitative review → R3-43 (unchanged) |
| 38 | Define, and differentiate between, concepts related to AI such as large language modeling (LLM), machine learning (ML), deep learning (DL), traditional AI, and generative AI. | 6.0 | 2.0 | Met consensus (median ≥ 6, IQR ≤ 2) — retained | Advanced → R3-18 (reworded after qualitative review) |
| 39 | Name the different types of AI technologies, specifically as they relate to medicine (e.g. large language model agents) | 6.0 | 2.0 | Met consensus (median ≥ 6, IQR ≤ 2) — retained | Advanced → R3-19 (unchanged) |
| 40 | Differentiate AI from technologies such as augmented reality (AR) and virtual reality (VR), specifically concerning their use in health care. | 6.0 | 2.0 | Met consensus (median ≥ 6, IQR ≤ 2) — retained | Advanced → R3-48 (reworded after qualitative review) |
| 41 | Select the most appropriate AI technology for the specific task or need at hand. | 6.0 | 1.5 | Met consensus (median ≥ 6, IQR ≤ 2) — retained | Advanced → R3-20 (reworded after qualitative review) |
| 42 | Describe how AI can support, and undermine, learning (as a medical student), including in areas such as critical thinking. | 6.0 | 1.5 | Met consensus (median ≥ 6, IQR ≤ 2) — retained | Advanced → R3-14 (reworded after qualitative review) |
| 43 | Use effective prompts when using generative AI for learning. | 6.0 | 1.5 | Met consensus (median ≥ 6, IQR ≤ 2) — retained | Merged into R3-13 after qualitative review |
| 44 | Use AI to support learning with simulated patients | 6.0 | 2.0 | Met consensus (median ≥ 6, IQR ≤ 2) — retained | Merged into R3-1 after qualitative review |
| 45 | Describe ethical challenges and limitations posed by AI in medical research and strategies to mitigate these. | 6.0 | 2.5 | High median but dispersed (IQR > 2) — qualitative review | Retained after qualitative review → R3-5 (reworded) |
| 46 | Evaluate clinical applications of AI | 6.0 | 1.0 | Met consensus (median ≥ 6, IQR ≤ 2) — retained | Advanced → R3-22 (reworded after qualitative review) |
| 47 | Implement strategies to avoid risks and pitfalls related to the use of AI in clinical practice. | 6.0 | 2.0 | Met consensus (median ≥ 6, IQR ≤ 2) — retained | Advanced → R3-24 (reworded after qualitative review) |
| 48 | Adapt to new AI technologies and methodologies as they emerge | 6.0 | 1.0 | Met consensus (median ≥ 6, IQR ≤ 2) — retained | Advanced → R3-41 (unchanged) |
| 49 | Maintain an ongoing commitment to learn about new AI tools, technologies and practices that emerge | 6.0 | 1.0 | Met consensus (median ≥ 6, IQR ≤ 2) — retained | Advanced → R3-40 (reworded after qualitative review) |
| 50 | Collaborate effectively with AI systems in clinical workflows | 6.0 | 1.0 | Met consensus (median ≥ 6, IQR ≤ 2) — retained | Advanced → R3-25 (unchanged) |
| 51 | Describe the application of AI technologies in clinical settings, including their roles in diagnostics, treatment planning and recommendations, patient monitoring, and risk assessment. | 6.0 | 1.5 | Met consensus (median ≥ 6, IQR ≤ 2) — retained | Advanced → R3-26 (reworded after qualitative review) |
| 52 | Practice transparency and professionalism by acknowledging the use of AI generated sources of information in medical education, research, and clinical practice. | 6.0 | 1.5 | Met consensus (median ≥ 6, IQR ≤ 2) — retained | Advanced → R3-9 (reworded after qualitative review) |
| 53 | Use AI effectively to stay up to date with the literature. | 5.5 | 2.8 | Below consensus (median < 6) — low priority | Advanced → R3-42 (unchanged) despite low-priority label — reinstated via qualitative review |
| 54 | Describe the use of AI, including generative AI, to support staying up to date with the literature. | 5.0 | 1.0 | Below consensus (median < 6) — low priority | Excluded (low priority); concept subsumed by R3-42 after qualitative review |
| 55 | Stay updated on the rapid evolution and advancement in AI technologies in health care, including clinical practice, learning and health services management. | 5.0 | 1.0 | Below consensus (median < 6) — low priority | Advanced → R3-39 Retained after qualitative review and reworded |
| 56 | Apply techniques and strategies to enhance the use of AI technologies and mitigate their limitations. | 5.0 | 2.0 | Below consensus (median < 6) — low priority | Excluded (low priority); after qualitative review subsumed by R3-17 |
| 57 | Participate in quality improvement, including incident reporting and analysis, related to the use of AI in clinical care. | 5.0 | 1.5 | Below consensus (median < 6) — low priority | Excluded (low priority) |
| 58 | Evaluate the quality of data derived from large data, especially in the context of quality improvement and research. | 5.0 | 2.5 | Below consensus (median < 6) — low priority | Advanced → R3-34 Retained after qualitative review and reworded |
| 59 | Describe regulatory guidelines governing AI in healthcare (HIPPA, GDPR) | 5.0 | 1.5 | Below consensus (median < 6) — low priority | Excluded (low priority); concept partially covered by R3-7 |
| 60 | Use AI to enhance the critical appraisal of literature (studies) | 5.0 | 1.5 | Below consensus (median < 6) — low priority | Advanced → R3-32 Retained after qualitative review (unchanged) |
| 61 | Summarize how AI algorithms work | 5.0 | 1.0 | Below consensus (median < 6) — low priority | Excluded (low priority) |
| 62 | Use generative AI effectively and professionally in case-based and problem-based learning. | 5.0 | 2.0 | Below consensus (median < 6) — low priority | Excluded (low priority) |
| 63 | Evaluate the strengths and limitations of AI systems in clinical settings | 5.0 | 2.5 | Below consensus (median < 6) — low priority | Excluded (low priority); concept overlaps R3-37/R3-45 |
| 64 | Interpret AI-generated outputs and integrate them into clinical decision-making | 5.0 | 2.0 | Below consensus (median < 6) — low priority | Excluded (low priority) |
| 65 | Describe the use of AI technologies in support of clinical workflows (such as documenting patient visits in electronic health records optimizing clinical workflow, etc.) | 5.0 | 2.0 | Below consensus (median < 6) — low priority | Advanced → R3-47 Retained after qualitative review and reworded |
| 66 | Describe the regulatory landscape governing the development and use of AI technologies in healthcare | 5.0 | 1.5 | Below consensus (median < 6) — low priority | Excluded (low priority); concept partially covered by R3-7 |
| 67 | Adapt to changes in practice workflows due to AI integration | 5.0 | 1.5 | Below consensus (median < 6) — low priority | Excluded (low priority) |
| 68 | Evaluate the quality (including validity, reliability, sensitivity, specificity, generalizability, and applicability) of studies on the use of AI in medicine | 5.0 | 2.0 | Below consensus (median < 6) — low priority | Excluded (low priority) |
| 69 | Explain how AI can help with operational efficiency in administrative tasks such as scheduling patients, maintaining medical records, searching medical records, and writing medical notes | 5.0 | 2.0 | Below consensus (median < 6) — low priority | Excluded (low priority); concept partially covered by R3-47 |
| 70 | Explain AI-generated or enhanced processes and results to patients in a way that aligns with best communication practices | 5.0 | 1.5 | Below consensus (median < 6) — low priority | Excluded (low priority); reworded into R3-29 |
| 71 | Critically analyze AI algorithms and models for clinical relevance and generalizability | 4.0 | 1.0 | Below consensus (median < 6) — low priority | Excluded (low priority) |
| 72 | Describe the process of providing input for AI model development and validation | 4.0 | 3.0 | Below consensus (median < 6) — low priority | Excluded (low priority) |
| 73 | Provide input for AI model development and validation | 4.0 | 2.0 | Below consensus (median < 6) — low priority | Excluded (low priority) |
| 74 | Apply data preprocessing for AI model development | 4.0 | 1.5 | Below consensus (median < 6) — low priority | Excluded (low priority) |
| 75 | Identify types of healthcare data | 4.0 | 2.5 | Below consensus (median < 6) — low priority | Excluded (low priority) |
| 76 | Describe the role of large data and data science in health care, and the use of AI technologies to support their applications. | 4.0 | 1.5 | Below consensus (median < 6) — low priority | Excluded (low priority) |
| 77 | Describe the historical evolution of AI | 3.0 | 2.0 | Below consensus (median < 6) — low priority | Excluded (low priority) |

## **Table S3. Round 3 (October 2024): ratings and disposition of the 48 retained learning objectives**

*Ratings by the panel (16 of 16 responded; 100%). The same consensus rule was applied unchanged. After qualitative review, merging and rewording, the list was reduced to 35 LOs organized under 6 overarching competencies for external validation.*

| **#** | **Learning objective (as rated)** | **Median** | **IQR** | **Disposition** | **Traceability: disposition after Round 3** |
| --- | --- | --- | --- | --- | --- |
| 1 | Use AI effectively, professionally, and ethically to support medical learning in UGME. (undergraduate medical education) | 7.0 | 1.0 | Met consensus (median ≥ 6, IQR ≤ 2) — retained | Elevated to the title of Competency 2 |
| 2 | Identify the limits of AI in support of learning (when should we use it and when should we not use it.) | 7.0 | 1.0 | Met consensus (median ≥ 6, IQR ≤ 2) — retained | Merged with R3-14 after qualitative review → final LO 2.1 |
| 3 | Describe how AI could enhance or undermine clinical reasoning. | 7.0 | 1.0 | Met consensus (median ≥ 6, IQR ≤ 2) — retained | Advanced → final LO 3.1 (expanded with examples) |
| 4 | Learn how to apply critical thinking when using and applying AI output in practice. | 7.0 | 0.0 | Met consensus (median ≥ 6, IQR ≤ 2) — retained | Split → final LOs 3.2 and 4.5 after qualitative review |
| 5 | Describe the ethical challenges and limitations of AI in medical research and strategies to mitigate these. | 7.0 | 1.0 | Met consensus (median ≥ 6, IQR ≤ 2) — retained | Advanced → final LO 4.9 (reworded) after qualitative review |
| 6 | Apply a multidisciplinary approach when adopting or using AI in clinical practice, including collaborating with data scientists, computer engineers, biomedical engineers, and other experts. | 7.0 | 1.0 | Met consensus (median ≥ 6, IQR ≤ 2) — retained | Advanced → final LO 6.2 (reworded after qualitative review) |
| 7 | Describe best practices and regulatory requirements for data protection, privacy, anonymity, and consent associated with using AI in healthcare. | 7.0 | 1.0 | Met consensus (median ≥ 6, IQR ≤ 2) — retained | Advanced → final LO 5.4 (reworded after qualitative review) |
| 8 | Use AI technologies ethically and professionally to support learning in medical education, clinical care, administration, research, and quality improvement. | 7.0 | 1.0 | Met consensus (median ≥ 6, IQR ≤ 2) — retained | Merged with R3-36 after qualitative review → final LO 5.1 |
| 9 | Describe under which circumstances healthcare professionals must practice transparency and professionalism by acknowledging the use of AI-generated information sources in medical education, research, and clinical practice. | 7.0 | 1.0 | Met consensus (median ≥ 6, IQR ≤ 2) — retained | Advanced → final LO 5.6 (reworded after qualitative review) |
| 10 | Apply AI technologies effectively when they are used to aid in clinical care, including analyzing results and making decisions. | 7.0 | 1.3 | Met consensus (median ≥ 6, IQR ≤ 2) — retained | Merged after qualitative review into final LO 4.5 |
| 11 | Practice in a person-centered, humanistic way (empathy, compassion, person-centeredness) in the context of extensive use of AI and other technologies. | 7.0 | 0.3 | Met consensus (median ≥ 6, IQR ≤ 2) — retained | Advanced → final LO 5.7 (reworded after qualitative review) |
| 12 | Recognize errors and biases within AI-derived information. | 7.0 | 0.3 | Met consensus (median ≥ 6, IQR ≤ 2) — retained | Advanced → final LO 2.3 (unchanged) |
| 13 | Create effective prompts for ChatGPT or other AI-generative platforms. | 6.5 | 1.3 | Met consensus (median ≥ 6, IQR ≤ 2) — retained | Advanced → final LO 2.2 (expanded after qualitative review) |
| 14 | Describe how AI can support and undermine learning (as a medical student) in general. | 6.5 | 1.3 | Met consensus (median ≥ 6, IQR ≤ 2) — retained | Merged with R3-2 after qualitative review → final LO 2.1 |
| 15 | Respond to patients in a way that demonstrates collaborative, shared decision-making and best evidence-driven care when patients approach with information they found using AI. | 6.5 | 1.0 | Met consensus (median ≥ 6, IQR ≤ 2) — retained | Advanced → final LO 4.6 (unchanged) |
| 16 | Explain the legal implications of AI usage, including potential risks. | 6.5 | 1.3 | Met consensus (median ≥ 6, IQR ≤ 2) — retained | Advanced → final LO 5.3 (reworded after qualitative review) |
| 17 | Implement strategies to mitigate risks and the use of AI technology in medical education. | 6.5 | 1.3 | Met consensus (median ≥ 6, IQR ≤ 2) — retained | Advanced → final LO 3.6 (expanded after qualitative review) |
| 18 | Define and differentiate between concepts related to AI, such as traditional AI, machine learning (ML), deep learning (DL), large language modeling (LLM), and generative AI. | 6.0 | 1.0 | Met consensus (median ≥ 6, IQR ≤ 2) — retained | Advanced → final LO 1.3 (expanded after qualitative review) |
| 19 | Name the different types of AI technologies, specifically as they relate to medicine (e.g. large language model agents) | 6.0 | 2.0 | Met consensus (median ≥ 6, IQR ≤ 2) — retained | Reworded after qualitative review into final LO 1.3 |
| 20 | Select the most appropriate AI technology for the specific task or need. | 6.0 | 1.0 | Met consensus (median ≥ 6, IQR ≤ 2) — retained | Advanced → final LO 2.4 (reworded after qualitative review) |
| 21 | Provide examples of the applications of AI in the various fields of medicine (e.g. clinical diagnostics and decision-making, health records management, screening, education, research). | 6.0 | 1.3 | Met consensus (median ≥ 6, IQR ≤ 2) — retained | Advanced → final LO 4.1 (unchanged) |
| 22 | Describe clinical applications of AI. | 6.0 | 1.3 | Met consensus (median ≥ 6, IQR ≤ 2) — retained | Reworded after qualitative review into final LO 4.4 |
| 23 | Describe ethical risks or pitfalls related to using AI in clinical practice. | 6.0 | 1.0 | Met consensus (median ≥ 6, IQR ≤ 2) — retained | Advanced → final LO 5.2 (unchanged) |
| 24 | Describe strategies to avoid risks and pitfalls related to using AI in clinical practice. | 6.0 | 1.0 | Met consensus (median ≥ 6, IQR ≤ 2) — retained | Advanced → final LO 4.2 (unchanged) |
| 25 | Collaborate effectively with AI systems in clinical workflows. | 6.0 | 1.0 | Met consensus (median ≥ 6, IQR ≤ 2) — retained | Reworded after qualitative review → final LO 4.3 |
| 26 | Describe the application of AI technologies in clinical settings, including their roles in diagnostics, treatment planning, patient monitoring, and risk assessment. | 6.0 | 2.0 | Met consensus (median ≥ 6, IQR ≤ 2) — retained | Advanced → final LO 4.4 (unchanged) |
| 27 | Use information and data derived from AI in combination with other reliable sources of medical information to aid in clinical care and learning. | 6.0 | 2.0 | Met consensus (median ≥ 6, IQR ≤ 2) — retained | Merged with R3 -28 into final LO 4.5 after qualitative review |
| 28 | Analyze information and data obtained by AI in healthcare. | 6.0 | 2.0 | Met consensus (median ≥ 6, IQR ≤ 2) — retained | Merged with R3-28 after qualitative review into final LO 4.5 |
| 29 | Inform patients and care providers about the use of AI in health care, including its strengths and limitations, when such technology is used to support their care (e.g., helping make care decisions). | 6.0 | 2.0 | Met consensus (median ≥ 6, IQR ≤ 2) — retained | Advanced → final LO 5.8 (reworded) |
| 30 | Describe the effective and ethical use of AI in medical research. | 6.0 | 1.3 | Met consensus (median ≥ 6, IQR ≤ 2) — retained | Advanced after qualitative review → final LO 4.7 (reworded) |
| 31 | Identify the opportunities and threats of the use of AI in medical research | 6.0 | 1.3 | Met consensus (median ≥ 6, IQR ≤ 2) — retained | Merged after qualitative review into final LO 4.9 |
| 32 | Use AI to enhance the critical appraisal of literature (studies) | 6.0 | 1.5 | Met consensus (median ≥ 6, IQR ≤ 2) — retained | Advanced → final LO 4.8 (expanded) |
| 33 | Describe AI technology to support quality improvement using electronic health records. | 6.0 | 1.3 | Met consensus (median ≥ 6, IQR ≤ 2) — retained | Advanced → final LO 4.10 (reworded after qualitative review) |
| 34 | Describe the quality of data derived from large data, especially in the context of quality improvement and research. | 6.0 | 1.0 | Met consensus (median ≥ 6, IQR ≤ 2) — retained | Advanced → final LO 4.11 (reworded after qualitative review) |
| 35 | Participate in and contribute medical expertise to interdisciplinary AI projects on health care delivery and health-related education. | 6.0 | 1.3 | Met consensus (median ≥ 6, IQR ≤ 2) — retained | Advanced → final LO 6.1 (reworded after qualitative review) |
| 36 | Identify ethical considerations when using AI technologies for learning, medical practice, health records keeping and analyses, research, and quality improvement. | 6.0 | 1.0 | Met consensus (median ≥ 6, IQR ≤ 2) — retained | Merged with R3-8 after qualitative review → final LO 5.1 |
| 37 | Explain the strengths and weaknesses of AI technologies used in health care. | 6.0 | 2.0 | Met consensus (median ≥ 6, IQR ≤ 2) — retained | Advanced → final LO 4.12 (unchanged) |
| 38 | Predict and prepare for potential opportunities and threats that AI technologies may create. | 6.0 | 1.5 | Met consensus (median ≥ 6, IQR ≤ 2) — retained | Advanced → final LO 3.5 (reworded after qualitative review) |
| 39 | Stay updated on AI technologies' rapid evolution and advancement in health care, including clinical practice, learning, and health services management. | 6.0 | 1.0 | Met consensus (median ≥ 6, IQR ≤ 2) — retained | Merged with R3-40 after qualitative review → final LO 7.1 |
| 40 | Maintain an ongoing commitment to learning about emerging AI tools, technologies, and practices. | 6.0 | 1.3 | Met consensus (median ≥ 6, IQR ≤ 2) — retained | Merged with R3-39 after qualitative review → final LO 7.1 |
| 41 | Adapt to new AI technologies and methodologies as they emerge. | 6.0 | 3.0 | High median but dispersed (IQR > 2) — qualitative review | Retained after qualitative review → final LO 7.2 |
| 42 | Use AI effectively to stay up to date with the literature. | 6.0 | 1.3 | Met consensus (median ≥ 6, IQR ≤ 2) — retained | Merged after qualitative review into final LO 4.8 |
| 43 | Define what AI is. | 5.5 | 3.0 | Below consensus (median < 6) — low priority – qualitative review | Reinstated after qualitative review → final LO 1.1 despite below-consensus rating |
| 44 | Describe AI technology in support of medical record keeping and retrieval of medical records. | 5.5 | 1.3 | Below consensus (median < 6) — low priority | Excluded (low priority); concept partially covered by final LO 4.3 |
| 45 | Explain the strengths and weaknesses of AI technologies used in medical education. | 5.5 | 2.0 | Below consensus (median < 6) — low priority | Reinstated after qualitative review → final LO 3.4 |
| 46 | Describe an overview of how AI systems are “trained” (e.g., supervised, unsupervised, and reinforcement learning). | 5.0 | 3.0 | Below consensus (median < 6) — low priority | Reinstated after qualitative review and expanded → final LO 1.2 |
| 47 | Describe the use of AI technologies to support clinical workflows (such as documenting patient visits in electronic health records, optimizing clinical workflow, etc.) | 5.0 | 1.3 | Below consensus (median < 6) — low priority | Reinstated/subsumed after qualitative review → final LO 4.3 |
| 48 | Differentiate AI from augmented reality (AR) and virtual reality (VR) regarding their use in health care. | 4.0 | 2.0 | Below consensus (median < 6) — low priority | Excluded as a standalone LO; AR/VR concepts subsumed into final LO 1.3 after qualitative review |

## **Table S4. Round 4 (November 2024–January 2025): external expert validation and final wording**

*Summary of the external validation round and the final team consensus meeting.*

| **Input to Round 4** | 35 learning objectives organized under 6 overarching competencies (the framework sent to the expert panel) |
| --- | --- |
| **Expert panel** | 10 external experts (medical educators and an AI/medical researcher) from Spain, the United States, Canada, Chile and Argentina; agree / agree-with-suggestions / disagree scale with free-text comments; 100% of the convened panel responded |
| **Competency-level outcome** | Unanimous agreement that the competencies were appropriate; 2 competencies reworded for clarity and 1 new competency added → 7 competencies |
| **LO-level outcome** | 20 LOs accepted unchanged; 15 received suggestions (rewording / merging / additions); 4 new LOs proposed by experts and 2 existing LOs merged owing to overlap → net +2 → 37 LOs |
| **Final consensus meeting** | The full team reviewed each competency, LO and expert suggestion and agreed the final wording by consensus (and the recommended year of study for each LO) |
| **Output of Round 4** | Final framework of 7 competencies and 37 supporting learning objectives |
