# Supplementary material for: What and when: a Delphi study identifying AI competencies and learning objectives for medical students
Source: Front Digit Health. 2026 Sep 30;8:1918847. doi: 10.3389/fdgth.2026.1918847 (PMC13662149; doi:10.3389/fdgth.2026.1918847)
Supplement: Supplementary file 7 [file Supplementaryfile7.docx]

Supplement 7 -Mapping the seven AI Competencies to CanMEDS roles and ACGME core competencies

| **Competency** | **CanMEDS role(s)** | **ACGME core competencies** |
| --- | --- | --- |
| 1. Define and describe AI. | Scholar | Medical Knowledge |
| 2. Use AI effectively, responsibly, and professionally to support learning in medicine. | Scholar; Professional | Practice-Based Learning and Improvement; Professionalism |
| 3. Critically appraise the potential strengths, limitations, risks and bias of AI in medical education and healthcare. | Scholar; Medical Expert, Leader, Communicator | Practice-Based Learning and Improvement; Medical Knowledge |
| 4. Explain the role of AI in supporting clinical care, research and quality improvement. | Medical Expert; Leader; Scholar | Patient Care; Systems-Based Practice |
| 5. Identify and apply best ethical and legal practices when using AI to support learning and clinical care. | Professional; Communicator; Medical expert | Professionalism; Interpersonal and Communication Skills |
| 6. Value the role of interdisciplinary teamwork in AI-related initiatives and projects. | Collaborator | Interpersonal and Communication Skills; Systems-Based Practice |
| 7. Demonstrate curiosity and awareness of emerging AI technologies, trends and applications. | Scholar; Professional | Practice-Based Learning and Improvement |
